# Centrality-based pathway enrichment: a systematic approach for finding significant pathways dominated by key genes

**DOI:** 10.1186/1752-0509-6-56

**Published:** 2012-06-06

**Authors:** Zuguang Gu, Jialin Liu, Kunming Cao, Junfeng Zhang, Jin Wang

**Affiliations:** 1 The State Key Laboratory of Pharmaceutical Biotechnology and Jiangsu Engineering Research Center for MicroRNA Biology and Biotechnology, School of Life Science, Nanjing University, Nanjing, 210093, China

**Keywords:** Pathway enrichment, Biological network, Centrality, Gene expression data

## Abstract

**Background:**

Biological pathways are important for understanding biological mechanisms. Thus, finding important pathways that underlie biological problems helps researchers to focus on the most relevant sets of genes. Pathways resemble networks with complicated structures, but most of the existing pathway enrichment tools ignore topological information embedded within pathways, which limits their applicability.

**Results:**

A systematic and extensible pathway enrichment method in which nodes are weighted by network centrality was proposed. We demonstrate how choice of pathway structure and centrality measurement, as well as the presence of key genes, affects pathway significance. We emphasize two improvements of our method over current methods. First, allowing for the diversity of genes’ characters and the difficulty of covering gene importance from all aspects, we set centrality as an optional parameter in the model. Second, nodes rather than genes form the basic unit of pathways, such that one node can be composed of several genes and one gene may reside in different nodes. By comparing our methodology to the original enrichment method using both simulation data and real-world data, we demonstrate the efficacy of our method in finding new pathways from biological perspective.

**Conclusions:**

Our method can benefit the systematic analysis of biological pathways and help to extract more meaningful information from gene expression data. The algorithm has been implemented as an R package CePa, and also a web-based version of CePa is provided.

## Background

As omics and high throughput technology continues to develop, researchers can increasingly understand biological phenomena at the systems level; that is, can elucidate the complicated interactions between genes and molecules responsible for biological functions [1]. Microarray technology has been widely used to measure gene expression profiles and has produced huge amounts of data for biological analysis [2]. However, traditional single gene analysis tells us little about the cooperative roles of genes in real biological systems. New challenges for microarray data analysis are to find specific biological functions affected by a group of related genes. Biological pathways are sets of genes or molecules that act together by chemical reactions, molecule modifications or signalling transduction to carry out such functions [3]. Since pathways are essentially integrated circuits that actualize specified biological processes, perturbation of pathways may be harmful to regular biological systems. Thus, finding biologically important pathways can assist researchers in identifying sets of genes responsible for essential functions. Currently, amount of tools are available to identify which pathways are significantly influenced based on the transcription level change of member genes [4,5]. In other words, they identify pathways where differentially expressed genes are enriched.

Since a pathway can be denoted as a set of genes, pathway enrichment belongs to a more general category of methods termed gene set enrichment. Two main categories of enrichment methodologies exist: over representation analysis (ORA) and gene set analysis (GSA) [6]. The former only focuses on the number of differential genes in the pathway, while the latter incorporates the entire gene expression from microarray datasets. In fact, ORA is a special case of GSA, utilizing a binary transformation of gene expression values. In standard ORA, the correlations between genes within the pathway and those that are differentially expressed are evaluated by Fisher’s exact test or chi-square test, in form of a 2 × 2 contingency table [7]. The most popular ORA online tool in current use is DAVID [8], which supports a variety of species and gene identifiers. For researchers familiar with the R statistical environment, the GOstats package [9] is a highly recommended ORA analysis tool. GSA methods are implemented via either a univariate or a multivariate procedure [6]. In univariate analysis, gene level statistics are initially calculated from fold changes or statistical tests (e.g., *t*-test). These statistics are then combined into a pathway level statistic by summation or averaging [6]. GSEA [10] is a widely used univariate tool that utilizes a weighted Kolmogorov-Smirnov test to measure the degree of differential expression of a gene set by calculating a running sum from the top of a ranked gene list. Multivariate analysis considers the correlations between genes in the pathway and calculates the pathway level statistic directly from the expression value matrix using Hotelling’s *T*^2^ test [11] or MANOVA models [12]. Besides these standard models, extended models of GSA exist. For example, GSCA (Gene Set Co-Expression Analysis) [13] aims to identify gene sets whose members have different co-expression structures between phenotypes; ROAST [14] uses a Monte-Carlo simulation for multivariate regression which is applicable to diverse experimental designs; GGEA (Gene Graph Enrichment Analysis) [15] evaluates gene sets as Petri networks constructed from an *a priori* established gene regulatory network. Further studies have focused on the methodology issues of gene set enrichment analysis, such as evaluating the power of different statistical models [6,16], generating null distributions of gene set scores [17,18], and overlapping of gene sets [19-21]. The approach of gene set enrichment analysis is also applicable to a broad range of systems-biology-related fields, including functional network module analysis [22] and microRNA target prediction [23,24].

Current enrichment methods are limited for pathway analysis because they treat genes identical in pathways. Rather than comprising a list of genes, a pathway identifies how member genes interact with each other. Clearly, perturbation on a key gene will make more considerable effect for the pathway than on an insignificant gene. Since a pathway is represented as a network with nodes and edges, its topology is essential for evaluating the importance of the pathway. To date, few pathway enrichment studies have incorporated any topological information. Gao *et al*. [25] designed a pathway score in which the values of all connected gene pairs are summed, where the value of a gene pair is obtained by multiplying the absolute normalized expression values of the paired genes. Hung *et al*. [26] defined a value for each gene based on the closest correlated neighbor genes, and assumed this value as the weight of the Kolmogorov-Smirnov test in GSEA procedure [10] for each pathway. Drăghici *et al*. [27] introduced a topology term into the scoring function, reflecting that the importance of genes is enhanced if they in turn influence important downstream genes. Thomas *et al*. [28] assigned larger weights to upstream and downstream pathway genes, and to genes having high connectivity, and then integrated into the maxmean statistics [29]. Currently available methods determine the importance of genes in the pathway by a single measure. However, because of the complexity of biological pathways and the varying characteristics of genes, such single-measure quantitation cannot fully capture the properties of different genes on biological environment. Thus, a model that comprehensively integrates both enrichment and topology information is urgently required.

Here, we propose a general, systematic and extensible enrichment methodology by which to find significant pathways using topology information. Two improvements of our method over current methods are apparent. First, given the diversity of genes’ characteristics and the difficulties of covering gene importance from all angles, we do not assume a fixed measurement for each gene but allow the user to specify the method by which network nodes will be weighted, as an optional parameter in the model. This feature enables researchers to assess gene importance from a perspective relevant to their particular biological problem. In our model, the importance of genes in pathways is assessed by network centralities. In graph theory, centrality provides a means of ranking nodes based on network structure. Different centrality measurements assign importance to nodes from different aspects. Degree centrality quantifies the number of neighbours to which a node directly connects, while betweenness defines the number of information streams passing through a given node. Generally speaking, large centrality values are assigned to central nodes in the network. Nodes representing metabolites, proteins or genes with high centralities are essential for maintaining biological networks in steady state [30,31]. Moreover, the relevance of a particular centrality measurement may vary according to the biological role of the pathway [32,33]. Choice of centrality measurement depends on the types of genes considered important in the pathway. Second, nodes rather than genes are taken as the basic units of pathways in the model. In general, the regular biological functions in significant pathways are usually altered where abnormal pathway states arise from abnormal internal node states. We note that pathway nodes may represent not only single genes, but also complexes and protein families. For a complex comprising more than one gene, if one member gene has been altered, the function of the whole complex is disrupted. On the other hand, a single gene may reside in multiple complexes; if this gene loses its function, all of its complexes will be influenced. Therefore a mapping procedure from genes to pathway nodes is applied in our model. The pathway nodes further include non-gene nodes such as microRNAs and compounds, which also contribute to the topology of the pathway. Hence, all types of nodes are retained in our pathway analysis.

In this article, the original pathway enrichment method is extended by assigning network centralities as node weights, and nodes are mapped from differentially expressed genes in pathways. The model is flexible in that it can readily accommodate available gene set enrichment methods and various topological measurements. Through a simulation study, we demonstrate how pathway significance depends on network structure and choice of centrality measurement. In the analysis of liver cancer data set, our model identified relevant biological processes which were bypassed using existing methods. We also demonstrate how key genes affect the significance of pathways directly underlying biological processes.

## Results and discussion

Because ORA methodology is easily implemented and rapidly executed, it is favored over GSA in applications [8]. Therefore, we focus mainly on the centrality-based extension of ORA, while the extension of GSA will be discussed briefly at the end of this article.

### Mapping genes to nodes

**Figure 1:**
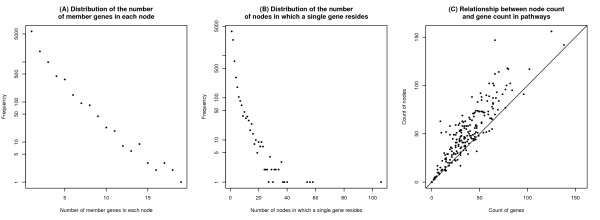
**Meta-analysis of the pathway catalogue. A)** Distribution of the number of member genes in each node; **B)** Distribution of the number of nodes in which a single gene resides; **C)** Relationship between node count and gene count in biological pathways. The pathways are derived from Pathway Interaction Database, NCI-Nature catalogue. For figure A and B, points are log-scaled on the Y-axis.

Since a pathway represents as a network, the basic unit of the network (the node) is not always a single gene. In real biological pathways, the nodes can also represent complexes or protein families. Moreover, the product of a particular gene may be incorporated into different complexes to serve different functions. Such diverse roles of gene products are ignored by traditional ORA methods, possibly leading to erroneous interpretations. Abnormal node states are expected to contribute to the abnormal states of pathways. As previously mentioned, the function of a multi-gene complex is affected by alteration of any one gene in the complex, while alteration of a multi-complex gene influences all of the complexes in which the gene resides. Merely counting genes in pathways cannot reflect these different types of roles played by different genes. In a real-world pathway catalogue, a node typically comprises two or more genes, and some genes locate in multiple complexes or families. Among pathways in the NCI-Nature catalogue of Pathway Interaction Database (PID) [34], 58.6% of nodes comprise more than one gene while 47.2% of genes reside in multiple nodes (Figure 1A, 1B). Compounds and microRNAs can also form pathway nodes. Although the changing quantity of these entities is not captured by typical microarray experiments, they may contribute significantly to pathway regulation. Therefore, these types of nodes cannot be neglected in topological pathway analysis. For the above reasons, the number of genes involved in a biological pathway does not correspond to the number of nodes in the pathway. Figure 1C shows how node count varies with gene count in pathways extracted from PID. Therefore, in our analysis we map genes to the pathway nodes and assume the node as the basic pathway unit. In this way, if any member of a complex or family is differentially expressed, the node representing the complex or family is differentially affected. We consider that nodes representing protein coding genes, compounds and microRNAs are all legitimate regulators of pathways.

### Pathway score

In any pathway enrichment framework, the significance of a pathway is evaluated by a pathway-level statistic. For example, in ORA, the pathway-level statistic is the number of differentially expressed genes in a pathway. To account for the varying positions of genes within pathways, we introduce a new statistic (here called the pathway score), defined as the summation of the weight of differentially affected nodes in the pathway:

(1) $$s=\sum_{i=1}^{n}w_{i}d_{i}$$

(2) $$d_{i}=\{\begin{array}{cc} 1 & \text{differentially affected}\\ 0 & \text{else} \end{array}$$

where *s* is the pathway score, *w*_*i*_ is the weight of the *i*^th^ node (reflecting the importance of the node), *n* is the number of nodes in the pathway, and *d*_*i*_ identifies whether the *i*^th^ node is differentially affected. The pathway score is the aggregate of two components, the weight and the number of differential nodes. Therefore, if a node has larger weight, i.e. is more important, it more strongly determines whether the pathway is significant. On the other hand, large numbers of differential nodes also increase the pathway score. Consequently, a significant pathway may contain a few highly important nodes, while an insignificant pathway contains many non-significant differential nodes. In Equation 1, the definition of *w* is general and the weight can be assigned any value the researcher considers appropriate. Note that when *w*_*i*_ = 1 for all *i*, *s* is simply the number of differential nodes in the pathway. We refer to this condition as the equal weight condition in the following section.

### Centrality measurements

The most important information in pathways comprises the complicated interactions between genes that govern the transmission of biological signals through networks. Since pathways present as networks, it is natural to define the weight *w* from topological information. In existing methods using topological information, various aspects of gene importance are assigned fixed values. It is noteworthy that, because genes play different roles in biological pathways, it is difficult to design measurements that cover the entire spectrum of a gene’s function. Instead of designing single measurements, we compute various topological measurements that measure the importance of genes from different aspects. Since different measurements relate to different biological functions, the best practice is to try every choice in the search for significant pathways.

Here, we identify central nodes in pathways using network centrality. Recall from the Background section that centrality in graph theory is a means of ranking nodes according to network structure. Two frequently-used centralities, degree and shortest path betweenness (or more concisely, betweenness), are selected as candidate measurements. Since pathways are directed networks, degree centrality is denoted as in-degree and out-degree. In biological networks, in-degree refers to the number of upstream genes directly acting on a given gene, while out-degree refers to the number of downstream genes directly acted upon by the gene. As previously mentioned, betweenness assesses the amount of information streaming through a given node in the network. These two centralities are broadly used in biological network analysis [31,35].

To measure the importance of nodes in the network from different aspects, we define an additional centrality: largest reach. The largest reach centrality is based on the shortest path between two nodes and is affected by all the other nodes in the network. The largest reach centrality determines how far a node can send or receive information within the network. It is defined as the largest length of the shortest paths to all the other nodes in the network. Since information is always transmitted sequentially in biological pathways, the largest reach centrality can reflect whether nodes stay in the upstream or downstream part of the pathway. In a directed network, the largest reach is denoted as in-largest reach and out-largest reach.

Other centralities, besides those described above, can also be considered. For instance, the closeness centrality computes the time required to spread information from one node to all other nodes. The eccentricity centrality determines whether a node resides in the center of the network and whether the distribution of nodes around the central node is symmetric. The stress centrality measures the extent to which a node can hold network communications. The eigenvector centrality measures the importance of a node based on its connections to other high-scoring nodes in the network (which contribute more to the node score than low-scoring nodes). Centralities closely related to the eigenvector are Katz’s Status Index and PageRank. For more details on this subject, readers may refer to [32,33,36].

### Simulation study

A novel gene list and a novel pathway are generated in the simulation study. In the pathway, we assume that every node corresponds to a single gene. The contingency table for ORA is listed in Table 1. The *p*-value of the pathway (1.36 × 10^−5^ by Fisher’s exact test, one sided) is constant and independent of pathway structure.

**Table 1:** 2 × 2 contingency table for ORA

	**In the pathway**	**Else**	**Total**
Differential	40	960	1000
Else	160	8840	9000
Total	200	9800	10000

The simulated microarray contains 10000 genes of which 1000 genes are differentially expressed. The novel pathway contains 200 genes of which 40 are differential genes.

The structure of the pathway is generated as random networks. Two representative random network models, Erdös-Rényi model [37] (abbreviated to ER) and Barabási-Albert model [38] (abbreviated to BA), are selected. These models are the basic random network models in graph theory but their network structures differ. We generate ER random networks as follows: 1) Each pair of nodes has the same probability (1/*n*) to be connected, where *n* is the number of nodes in the pathway; 2) Each connection can choose a direction with equal probability (*p* = 0.5). The BA random network is generated as follows: 1) The probability that a node will make a new connection is proportional to its degree; 2) Each connection can choose a direction with equal probability (*p* = 0.5). In the ER model, node degree follows a binomial distribution; while in BA model it follows a power law distribution. In the BA model, the majority of nodes have few neighbors while a small minority holds most connections in the network. Examples of ER and BA random networks can be found in Additional file 1.

The structure of the pathway was generated for 1000 times, and 40 differential nodes were randomly selected from each simulated network. For each simulated network, we calculate the significant of the pathway. Values of in-degree, out-degree, betweenness, in-largest reach, out-largest reach centralities, as well as the equal weight condition, are compared between our method and traditional ORA. Note that since every node corresponds to a single gene, the equal weight condition approximates to the hypergeometric distribution, on which traditional ORA is based [7].

**Figure 2:**
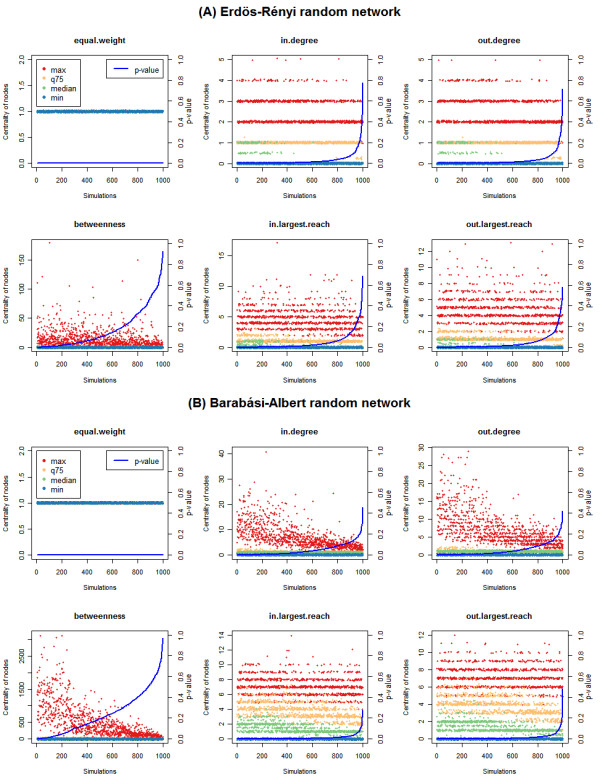
***P*-values and centrality distributions of pathways with different random network structures under different centrality measurements.** Pathway topologies are generated from **(A)** Erdös-Rényi model and **(B)** Barabási-Albert model. Comparisons are made between in-degree, out-degree, betweenness, in-largest reach, out-largest reach centralities, as well as the equal weight condition. Each plot represents the distribution of differential nodes centralities in each simulation, assessed by maximum value, the 75th quartile, median value and minimum value. All data are ordered by *p*-values on the X-axis. Points in the figure are randomly shifted by small intervals for ease of visualization.

Since the pathway score is computed from a list of differential nodes, we measure the approximate distribution of the differential nodes’ centrality in each simulation by four values: maximum, median, minimum and 75^th^ quartile. From these four values, the effect of the differential nodes’ centralities on the final pathway score can be estimated. Figure 2 illustrates *p*-values and distribution of centralities of differential nodes in each simulation under different centrality measurements. The proportions of the pathway with *p*-values ≤ 0.01 are listed in Table 2. Clearly, the significance of the pathway is lost when centrality is used as a weighting factor, and levels of pathway significance depend on network structure and type of centrality measure. For example, in an ER-generated network structure in which nodes are weighted by in-degree, the proportion of being significant for the pathway is 57.4% out of 1000 simulations.

**Table 2:** **Proportion of pathways with*p*-values ≤ 0.01 in simulation study**

**Centrality**	**ER model**	**BA model**
Equal weight	1.000	1.000
In-degree	0.574	0.383
Out-degree	0.574	0.403
Betweenness	0.134	0.081
In-largest reach	0.493	0.767
Out-largest reach	0.448	0.745

When using degree (in and out) as the weight, the ER model outputs a larger proportion of significant pathways than does the BA model. In BA, a small minority of important nodes (measured by degree) dominates the pathway; hence, if differential nodes are randomly picked from a BA network, the probability of selecting those nodes which yield large pathway scores is low. The majority of trials, therefore, generate insignificant pathways.

It is observed that maximum largest reaches (in and out) from both ER and BA networks are similar (around 10; see Figure 2), but the median values and the 75^th^ quartile of largest reach in the BA-generated network exceed those of the ER-generated network, implying that the distribution of largest reach in BA model is right shifted relative to that of the ER model (The histograms of the largest reach in both models can be found in Additional file 2). As a result, when using largest reach as weight, the BA model produces a higher proportion of significant pathways than does the ER model. This is due to the presence of central hub nodes in the BA model, which ensure robust information transmission and are thus more likely to score high largest reach values.

From the simulation study, we observe that although the number of differential nodes in a pathway is significant by Fisher’s exact test (or by its approximation, the equal weight condition), the pathway will not be significantly affected if these genes hold less important positions in the pathway. The level of significance is affected by both centrality measurements and network structure. If researchers consider that nodes with large degree will be more important, without considering the network topology, traditional ORA would yield large false positives. In the current simulation study, the proportion of significant pathway under ORA is expected to be 100%; but, when the structure of the pathway is assembled by the ER model and assessed by degree centrality, there are only 57.4% significant pathways from 1000 simulations. It means there would be 42.6% false positives from above perspective.

### Influence of key nodes

We next assess the influence of the key nodes in the evaluation of pathway significance. For the same novel gene list and novel pathway as were used in the simulation study, the number of differential nodes in the pathway is varied from 1 to 100. The pathway structures are generated from the BA model with no directions, and degree is used as the centrality measure. Differential nodes may be integrated into the pathway via two approaches; 1) from largest to smallest degree, and 2) from smallest to largest degree.

**Figure 3:**
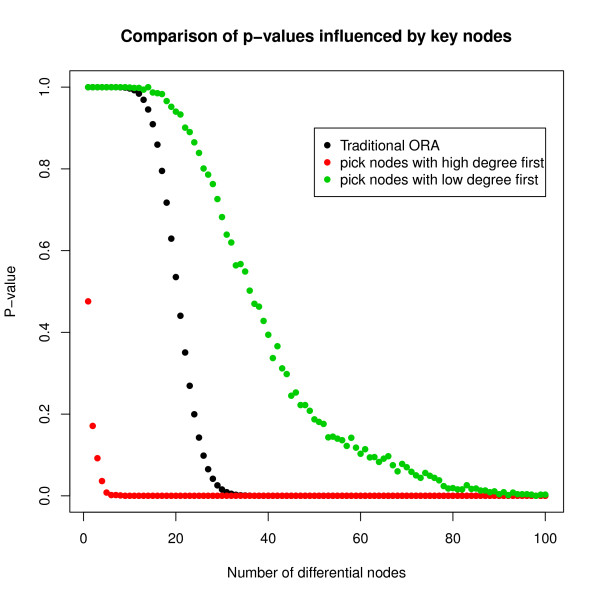
**Comparison of*p*-values influenced by key nodes.** Differential nodes, weighted by degree, are selected in two ways: from high to low degree and from low to high degree. Also, traditional ORA was applied for comparison.

In the BA model the small number of nodes holding most connections are the most central nodes, thus they contribute majorly to the significance of the pathway. The pathway would be altered if these nodes were differentially affected. As illustrated in Figure 3, when selecting high-degree differential nodes, provided that the number of differential nodes is 5 or greater, the pathway is highly significant (*p*-value < 0.01). By comparison, pathways generated from 5 differential nodes by traditional ORA are far from significant (*p*-value ≈ 1). Applying ORA, the minimum number of differential nodes required to achieve *p*-value < 0.01 is 31. On the contrary, if differential nodes in the pathway are largely of very low degree, many more of these nodes are required to make the pathway significant. As shown in Figure 3, at least 90 small-degree differential nodes must be selected to render the *p*-value of the pathway less than 0.01. In conclusion, considering the number of differential nodes alone cannot fully reflect the significance of the pathway. We reiterate that without highlighting these key nodes, researchers are likely to make erroneous interpretations of biological pathways.

### Real-world data analysis

We tested our method on a real microarray dataset [GEO: GSE22058] [39]. The microarray experiment measures mRNA expression changes in liver cancer tissue and adjacent non-tumour tissue. Following gene ID matching and duplicated gene merging, 18503 genes were obtained. The top 2000 most differentially expressed genes (determined by *t*-test) comprised our differential gene list. NCI-Nature pathway catalogue from Pathway Interaction Database (PID) [34] was used because it is manually curated and reviewed, and is highly recommended by the PID database. In-degree, out-degree, betweenness, in-largest reach and out-largest reach centrality measurements were applied and compared. In addition, we applied the dataset to equal weight condition and traditional ORA because the equal weight condition maps genes to nodes, while traditional ORA focuses solely on gene number. *P*-values for pathways are calculated from 1000 simulations and the false discovery rate (FDR) is calculated by Benjamini-Hochberg (BH) process [40]. Cutoff for FDR is set to 0.05.

**Figure 4:**
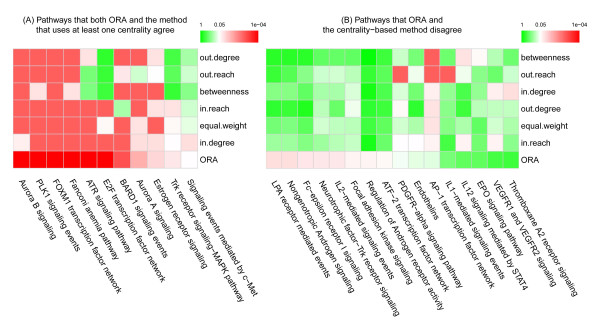
**Heatmap of FDRs in pathways. A)** Pathways evaluated as significant by both traditional ORA and our method for at least one centrality measure; **B)** Pathways for which our method disagrees with traditional ORA. In each heatmap, columns are sorted by FDRs calculated from traditional ORA and rows are sorted through hierarchical clustering. Green and red denote insignificant and highly significant, respectively.

**Figure 5:**
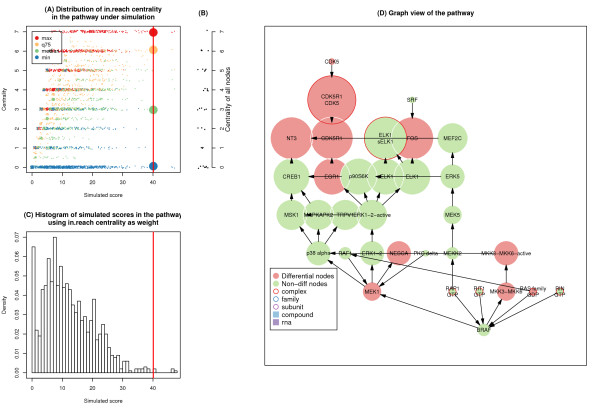
**Summary of MAPK-TRK pathway generated under in-largest reach centrality. A)** Distribution of in-largest reach centrality of differential nodes in the simulated pathway. The distribution of differential nodes centralities in each simulation is assessed by maximum value, the 75th quartile, median value and minimum value; **B)** Distribution of in-largest reach centrality of all nodes in the real pathway; **C)** Histogram of simulated scores in the pathway; **D)** Graph view of the pathway where the size of a node is proportional to its centrality value and nodes in red represent differential nodes. In figures A and B, dots are randomly shifted by small intervals for ease of visualization. In figures A and C, the real pathway score is marked with a red line.

Figure 4 illustrates the heatmaps of the FDRs of pathways generated under different centrality measurements. A complete list of *p*-values and FDRs is tabulated in Additional file 3 and Additional file 4. Among the 11 pathways for which our method agrees with traditional ORA using at least one centrality, the PLK pathway, MET pathway and MAPK pathway are directly related to liver cancers [41,42]. MAPK pathway is significant when nodes are weighted by in-largest reach (*p*-value = 0.001, FDR = 0.025), consistent with expected biological phenomena. The differential nodes are mainly located in the downstream of the pathway; that is, transcriptional factors (e.g. FOS) or cell cycle related factors (e.g. CDK5 and CD5R1), while few of the upstream genes are included in our differential gene list. As the MAPK pathway is essentially a cascade of sequential interactions [43], weighting its nodes by out-largest reach renders it insignificant, whereas weighting by in-largest reach, which gives larger weight to the downstream nodes, marks the pathway as significant (Figure 5). In other words, if the pathway is rendered significant by in-largest reach weighting, we can infer that the downstream nodes are differentially affected.

Among 8 pathways evaluated as insignificant by traditional ORA but significant by centrality-based methods, four have been previously linked to liver cancers [42,44,45]. AP-1 pathway is assessed as insignificant by traditional ORA because, of the 70 genes involved in the pathway, only 15 are differential. However, after mapping genes to the pathway nodes, we obtain 55 differential nodes among 114 pathway nodes. Because two key genes, FOS and JUN [46,47], combine with a host of other genes to form activated complexes in the pathway, the mapping procedure increases the number of positions that these two genes occupy in the network. Therefore the AP-1 pathway becomes more significant under equal weight condition than under traditional ORA. As another example, the VEGF receptor (VEGFA) is a principal component in the VEGFR1 and VEGFR2 signaling pathway. As a membrane protein, VEGFA receives large quantities of extracellular information and disseminates it into intracellular proteins [48]. VEGFA requiresVEGFR2 to form an activated complex, hence the representative node possesses high values of both in-degree and out-degree, and the degree-weighted pathway is rendered significant (*p*-value = 0.002, FDR = 0.034 for in-degree; *p*-value = 0.007, FDR = 0.104 for out-degree). On the other hand, VEGFA itself is not differentially expressed, but its companion gene VEGFR2 is. Consequently, an abnormal state of the member gene results in a dysfunctional complex. This type of circumstance, which cannot be inferred by traditional ORA, emphasizes why nodes, rather than genes, should form the basic units in pathway analysis.

## Conclusions

Pathway analysis can assist researchers to understand biological aberrations at a systems level. The functionality of biological pathways depends upon complex gene interactions. Therefore, pathway enrichment tools should highlight genes that play important roles in the pathway from the view of topology. Here we proposed a systematic and extensible methodology, which finds significant pathways using network centrality to weight the nodes. We demonstrated that levels of pathway significance depend on choice of pathway structure and centrality measure. The method performed favorably when applied to real-world data.

Centrality can reflects the central nodes in a pathway, and different centralities assign gene importance from different aspects. The use of centralities in biological networks can aid in explaining biological phenomena. In this work, we demonstrated the advantages of using multiple centrality measurements to obtain a complete view of the system. Pathway nodes, rather than genes, should form the basic units in pathway analysis, since many genes must aggregate as complexes in order to function completely. The focus on pathway nodes accommodates the fact that genes can be members of complexes or families, or may exist in many complexes. Finally, it should be noted that a high quality and non-redundant pathway structure dataset is required. Projects like BioPAX [49], which aspire to the integration and exchange of biological pathway data, will greatly assist future pathway analysis.

Our method can reveal new findings that relate to, and can aid the understanding of, current biological problems. We consider that our method will become a valuable tool in the systematic analysis of biological pathways, and will help to extract more meaningful information from gene expression data.

## Methods

**Figure 6:**
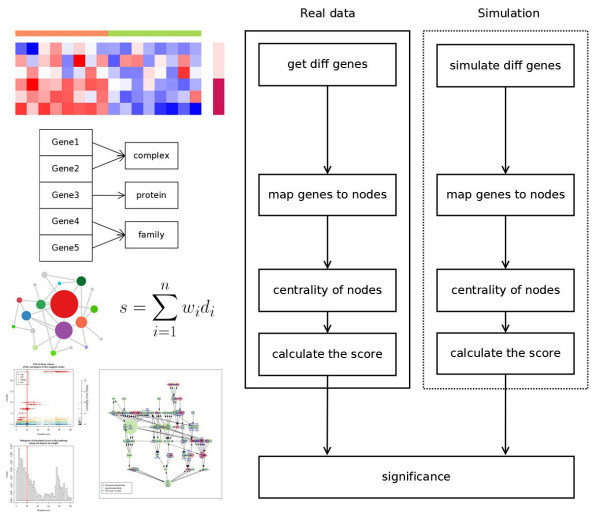
**Workflow of the centrality-based pathway enrichment analysis.** A typical figure on the left illustrates the corresponding step on the right side. The essential steps are: 1) Obtain a differentially expressed gene list. This list can be compiled using a variety of methods and sources; 2) Map genes to nodes; 3) Select several centrality measurements and calculate their values; 4) Weighting nodes by centrality, calculate the pathway-level score; 5) In simulations, repeat steps 1 to 4 for a user-specified number of cycles (1000 cycles were used in the current study) and generate a null distribution of pathway-level scores; 6) Calculate *p*-values and display the results summary.

To implement the method, a list of differential genes and a list of background genes, both formatted with gene identifier (e.g. gene symbol or RefSeq ID), is required. A list of pathways and their topology, and a means of mapping genes to pathway nodes, is also required. In this study, 223 NCI-Nature pathways from PID (released September 9^th^ 2011) are included. The pathway data are parsed from XML format file provided by the PID FTP site. The Perl code for parsing can be obtained from the author’s website (http://mcube.nju.edu.cn/jwang/lab/soft/cepa/). The general workflow of the method is illustrated in Figure 6.

### Generate mapping data

PID provides mappings from UniProt ID to node id. In this study, gene symbol is selected as the primary identifier ID. The mapping from gene symbol to HGNC ID [50] (accomplished via the online “custom downloads” tool in HGNC database) and the mapping from HGNC ID to UniProt ID [51] (using idmapping.dat.gz on the UniProt FTP site) are first extracted. The final mapping from gene symbol to node id is generated by merging the above three kinds of mapping data.

### Centrality measurements

Two commonly used centralities, degree and shortest path betweenness, are selected as initial candidate measurements. Degree centrality quantifies the number of neighboring nodes to which the node of interest is directly connected, while betweenness centrality measures the amount of information streaming through a given node.

To measure the importance of nodes in the network from multiple aspects, we defined an additional centrality: largest reach. This centrality is based on the shortest path between two nodes and the value of the centrality is affected by all other nodes in the network. The largest reach centrality measures how far a node can send or receive information. It is defined as the largest length of the shortest path from node *v* to all other nodes in the network (see Equations 3 and 4 where *d*(*w*, *v*) refers to the shortest path length between nodes *v* and *w*). In a directed network, this measure is denoted as in-largest reach or out-largest reach.

(3) $$C_{\mathrm{lr}}^{\mathrm{in}}(v)=\max_{w\in V}\left\{d(w,v)\right\}$$

(4) $$C_{\mathrm{lr}}^{\mathrm{out}}(v)=\max_{w\in V}\left\{d(v,w)\right\}$$

Users of our system can replace the provided centrality measures with their centrality measurements of interest. It is recommended that centrality choice is guided by biological plausibility and/or reality.

### Pathway score

The score is defined as the summation of the weights of differentially affected nodes in the pathway

(5) $$s=\sum_{i=1}^{n}w_{i}d_{i}$$

(6) $$d_{i}=\{\begin{array}{cc} 1 & \text{differentially affected}\\ 0 & \text{else} \end{array}$$

where *s* is the score of the pathway, *w*_*i*_ is the weight of the *i*^th^ node and reflects the importance of the node, *n* is the number of nodes in the pathway, and *d*_*i*_ identifies whether the *i*^th^ node is differentially affected or not.

In our model, we weight the nodes by network centrality. Because the network centrality can be zero, an additional term is added to the weight measure. In Equation 7, *α* is a small positive number to ensure that all weights are positive. *α* is chosen to exert marginal effect upon the weight. The default value of *α* is 1/100 of the minimum non-zero weight.

(7) $$w_{i}^{'}=w_{i}+\alpha$$

### Theoretical distribution of the pathway score

To calculate the theoretical distribution of the pathway score, we assume that every node is a single gene and that our model satisfies the following conditions: 1) genes are independent; 2) *w* and *d* are random variables; 3) *w* and *d* are independent; 4) *w* follows a particular discrete or continuous distribution and *d* is a Bernoulli random variable. Thus the pathway score, denoted as *S*, is also a random variable. These conditions are formally expressed as

(8) $$w\sim P_{w}(w)$$

(9) $$P(d=1)=1-P(d=0)=p_{\mathrm{diff}}$$

where *p*_*diff*_ is the probability that a gene is differentially expressed. It is calculated as the proportion of differentially expressed genes on the microarray.

(10) $$p_{\mathrm{diff}}=\frac{n_{\mathrm{diff}}}{n_{\mathrm{bg}}}$$

Within a pathway of score *s*, assume that *k* differential genes (*d* = 1) and *n*-*k* non-differential genes (*d* = 0) exist, so that *s* can be written as

(11) $$s=w_{1}+\ldots +w_{k}+0_{k+1}+\ldots +0_{n}$$

Thus the probability that pathway score *S* is equal to or larger than *s* is

(12) $$P(S\geq s)=\sum_{k=0}^{n}\left[\left(\begin{array}{c} n\\ k \end{array}\right)p_{\mathrm{diff}}^{k}{\left(1-p_{\mathrm{diff}}\right)}^{n-k}P_{w}\left(\sum_{i=1}^{k}w_{i}\geq s\right)\right]\text{.}$$

The binomial term of equation 12 is the probability of obtaining *k* differential genes from *n* genes, and the second term is the probability that the sum of *k* differential genes’ weight is equal to or larger than *s*. The final probability *P*(*S* ≥ *s*) is the summation over all conditions of *k*.

Since genes are independent, provided that *P*_*w*_(*w*) is known, the distribution of the summation of *w* can be calculated. For instance, given a pathway with ER random network structure in which nodes are weighted by degree, *w* will follow a binomial distribution and thus *P*(Σ_*i*_*w*_*i*_) also follows a binomial distribution.

### Non-parametric null distribution of the pathway score

In applications, because the weight distribution is not easily determined and nodes are not independent after the mapping procedure, the theoretic distribution is difficult to calculate. A non-parametric null distribution of *s* can be generated through simulation. For every gene in a pathway, we guess whether it is differentially expressed. Similar to throwing a coin, we assume that each gene has a probability *p*_*diff*_ (calculated by Equation 10) of being differentially expressed. In each simulation, we obtain a list of simulated differentially expressed genes in the pathway. This simulated differential gene list is then mapped to the pathway nodes. The pathway structure is unchanged and the simulated pathway score is re-calculated from Equations 5 and 6. The significance is calculated as the proportion of the simulated score exceeding the real score (Equation 13).

(13) $$p=\#\left\{s^{\mathrm{simulate}}\geq s\right\}/\#\{simulation\}$$

### Extension on GSA

The ORA centrality-based enrichment method yielded plausible, biologically relevant results in the simulation study and real-world data analysis. However, an oft-mentioned drawback of ORA is that an objective cutoff is appointed in the acquisition of a differential gene list, with the following consequences: 1) The resulting pathway or network may be sensitive to the cutoff [52]. In the centrality-based extension of ORA, when a high-scoring node is marginally close to the imposed cutoff, this effect can be critical; 2) In some circumstances, differential genes are too few to apply ORA [53]; 3) Binary transformation of expression data leads to loss of information. To address these issues, researchers have developed the GSA framework, which utilizes all gene expression values. Like traditional ORA however, GSA assumes that genes in pathways occupy unvarying positions in the topological structure. We propose that our centrality-based enrichment methodology can be similarly extended on GSA. In this section, we suggest, but do not implement, a conceptual methodological extension to the GSA method.

In the traditional univariate GSA procedure, the score *s* of the pathway is defined as:

(14) s=f(g)

where *f* transforms the gene-level statistic to a pathway-level statistic (e.g. by summation, averaging) and **g** is the gene-level statistic vector which typically comprises *t*-values [6,10,52]. In ORA, **g** is a binary variant and *f*(**g**) is summation. In our model to extend GSA, gene-level statistic is first transformed to node-level statistic. We define the vector of the node-level statistics as **d**. When nodes in pathways comprise multiple genes, the node-level statistic can be considered as the maximum value of the corresponding member genes. Using centrality as the weight, the score is defined as

(15) s=f(w·d)

where **w** is the weight vector and the transformation function *f* acts upon the product of **w** and **d**. Equation 15 incorporates centrality weight into the original node-level statistic. To prevent **w** from overpowering **d** (or vice versa) when both vectors contain continuous variables, we propose that **w** and **d** should be normalized. The null distribution of the pathway score could then be generated by permuting the gene expression matrix.

### Implementation

The method proposed in the article has been implemented in an R package named CePa which is available at CRAN (http://www.r-project.org/). In the CePa package, four pathway catalogues, namely NCI-Nature, BioCarta, Reactome and KEGG from PID, have been integrated. Centrality calculation and network visualization are processed by igraph package [54]. A web-based version of CePa is available for researchers who are not familiar with R programming (http://mcube.nju.edu.cn/cgi-bin/cepa/main.pl), in which Cytoscape Web is used for network visualization [55].

## Abbreviations

ORA, Over representation analysis; GSA: Gene set analysis; ER: Erdös-Rényi model; BA: Barabási-Albert model; PID: Pathway Interaction Database; BH: Bajamini-Hochberg process; FDR: False discovery rate.

## Competing interests

The authors declare that they have no competing interests.

## Authors’ contributions

ZG developed the algorithm, performed the analysis, implemented the software and wrote the manuscript. JL and KC implemented the online version of the software. JZ and JW conceived the study, and helped to draft the manuscript. All authors have read and approved the final manuscript.

## Supplementary Material

Additional file 1 **Two random pathways generated from ER model and BA model.** Number of nodes in both networks is 200. Click here for file

Additional file 2 Histograms of in-largest reach aggregated from 1000 networks generated either by ER model or BA model. Click here for file

Additional file 3 **The complete list of *p*-values of pathways generated under different centrality measurements.** Click here for file

Additional file 4 The complete list of FDRs of pathways generated under different centrality measurements. Click here for file

## References

[B1] Kitano H Systems biology: a brief overview Science 2002 295 1662 1664 10.1126/science.1069492 11872829

[B2] Allison DB Cui X Page GP Sabripour M Microarray data analysis: from disarray to consolidation and consensus Nat Rev Genet 2006 7 55 65 10.1038/nrg1749 16369572

[B3] Cary MP Bader GD Sander C Pathway information for systems biology FEBS lett 2005 579 1815 1820 10.1016/j.febslet.2005.02.005 15763557

[B4] Huang DW Sherman BT Lempicki RA Bioinformatics enrichment tools: paths toward the comprehensive functional analysis of large gene lists Nucleic Acids Res 2009 37 1 13 10.1093/nar/gkn923 19033363 PMC2615629

[B5] Khatri P Drăghici S Ontological analysis of gene expression data: current tools, limitations, and open problems Bioinformatics 2005 21 3587 3595 10.1093/bioinformatics/bti565 15994189 PMC2435250

[B6] Ackermann M Strimmer K A general modular framework for gene set enrichment analysis BMC bioinformatics 2009 10 47 10.1186/1471-2105-10-47 19192285 PMC2661051

[B7] Rivals I Personnaz L Taing L Potier MC Enrichment or depletion of a GO category within a class of genes: which test? Bioinformatics 2007 23 401 407 10.1093/bioinformatics/btl633 17182697

[B8] Huang DW Sherman BT Lempicki RA Systematic and integrative analysis of large gene lists using DAVID bioinformatics resources Nat Protoc 2009 4 44 57 19131956 10.1038/nprot.2008.211

[B9] Falcon S Gentleman R Using GOstats to test gene lists for GO term association Bioinformatics 2007 23 257 258 10.1093/bioinformatics/btl567 17098774

[B10] Subramanian A Tamayo P Mootha VK Mukherjee S Ebert BL Gillette MA Paulovich A Pomeroy SL Golub TR Lander ES Mesirov JP Gene set enrichment analysis: a knowledge-based approach for interpreting genome-wide expression profiles Proc Natl Acad Sci USA 2005 102 15545 15550 10.1073/pnas.0506580102 16199517 PMC1239896

[B11] Song S Black MA Microarray-based gene set analysis: a comparison of current methods BMC Bioinformatics 2008 9 502 10.1186/1471-2105-9-502 19038052 PMC2607289

[B12] Hummel M Meister R Mansmann U GlobalANCOVA: exploration and assessment of gene group effects Bioinformatics 2008 24 78 85 10.1093/bioinformatics/btm531 18024976

[B13] Choi Y Kendziorski C Statistical methods for gene set co-expression analysis Bioinformatics 2009 25 2780 2786 10.1093/bioinformatics/btp502 19689953 PMC2781749

[B14] Wu D Lim E Vaillant F Asselin-Labat ML Visvader JE Smyth GK ROAST: rotation gene set tests for complex microarray experiments Bioinformatics 2010 26 2176 2182 10.1093/bioinformatics/btq401 20610611 PMC2922896

[B15] Geistlinger L Csaba G Küffner R Mulder N Zimmer R From sets to graphs: towards a realistic enrichment analysis of transcriptomic systems Bioinformatics 2011 27 i366 i373 10.1093/bioinformatics/btr228 21685094 PMC3117393

[B16] Naeem H Zimmer R Tavakkolkhah P Küffner R Rigorous assessment of gene set enrichment tests Bioinformatics 2012 28 1408 1406 10.1093/bioinformatics/bts156 22492315

[B17] Goeman JJ Bühlmann P Analyzing gene expression data in terms of gene sets: methodological issues Bioinformatics 2007 23 980 987 10.1093/bioinformatics/btm051 17303618

[B18] Gatti DM Barry WT Nobel AB Rusyn I Wright FA Heading down the wrong pathway: on the influence of correlation within gene sets BMC Genomics 2010 11 574 10.1186/1471-2164-11-574 20955544 PMC3091509

[B19] Sohn I Owzar K Lim J George SL Mackey Cushman S Jung SH Multiple testing for gene sets from microarray experiments BMC Bioinformatics 2011 12 209 10.1186/1471-2105-12-209 21615889 PMC3131260

[B20] Newton MA He Q Kendziorski C A Model-Based Analysis to Infer the Functional Content of a Gene List Stat Appl Genet Mol Biol 2012 11 10.2202/1544-6115.1716 PMC3351831 22499692

[B21] Jiang Z Gentleman R Extensions to gene set enrichment. Bioinformatics 2007 23 306 313 10.1093/bioinformatics/btl599 17127676

[B22] Liu M Liberzon A Kong SW Lai WR Park PJ Kohane IS Kasif S Network-based analysis of affected biological processes in type 2 diabetes models PLoS Genet 2007 3 e96 10.1371/journal.pgen.0030096 17571924 PMC1904360

[B23] Creighton CJ Nagaraja AK Hanash SM Matzuk MM Gunaratne PH A bioinformatics tool for linking gene expression profiling results with public databases of microRNA target predictions RNA 2008 14 2290 2296 10.1261/rna.1188208 18812437 PMC2578856

[B24] Cheng C Li LM Inferring microRNA activities by combining gene expression with microRNA target prediction PloS One 2008 3 e1989 10.1371/journal.pone.0001989 18431476 PMC2291556

[B25] Gao S Wang X TAPPA: topological analysis of pathway phenotype association Bioinformatics 2007 23 3100 3102 10.1093/bioinformatics/btm460 17890270 PMC2473868

[B26] Hung JH Whitfield TW Yang TH Hu Z Weng Z DeLisi C Identification of functional modules that correlate with phenotypic difference: the influence of network topology Genome Biol 2010 11 R23 10.1186/gb-2010-11-2-r23 20187943 PMC2872883

[B27] Draghici S Khatri P Tarca AL Amin K Done A Voichita C Georgescu C Romero R A systems biology approach for pathway level analysis Genome Res 2007 17 1537 1545 10.1101/gr.6202607 17785539 PMC1987343

[B28] Thomas R Gohlke JM Stopper GF Parham FM Portier CJ Choosing the right path: enhancement of biologically relevant sets of genes or proteins using pathway structure Genome Biol 2009 10 R44 10.1186/gb-2009-10-4-r44 19393085 PMC2688935

[B29] Efron B Tibshirani R On testing the significance of sets of genes The Annals of Applied Statistics 2007 1 107 129 10.1214/07-AOAS101

[B30] Fell DA Wagner A The small world of metabolism Nat Biotechnol 2000 18 1121 1122 10.1038/81025 11062388

[B31] Jeong H Mason SP Barabási AL Oltvai ZN Lethality and centrality in protein networks Nature 2001 411 41 42 10.1038/35075138 11333967

[B32] Junker BH Koschützki D Schreiber F Exploration of biological network centralities with CentiBiN BMC Bioinformatics 2006 7 219 10.1186/1471-2105-7-219 16630347 PMC1524990

[B33] Scardoni G Petterlini M Laudanna C Analyzing biological network parameters with CentiScaPe Bioinformatics 2009 25 2857 2859 10.1093/bioinformatics/btp517 19729372 PMC2781755

[B34] Schaefer CF Anthony K Krupa S Buchoff J Day M Hannay T Buetow KH PID: the Pathway Interaction Database Nucleic Acids Res 2009 37 D674 D679 10.1093/nar/gkn653 18832364 PMC2686461

[B35] Joy MP Brock A Ingber DE Huang S High-betweenness proteins in the yeast protein interaction network J Biomed Biotechnol 2005 2005 96 103 10.1155/JBB.2005.96 16046814 PMC1184047

[B36] Koschützki D Schreiber F Centrality analysis methods for biological networks and their application to gene regulatory networks Gene Regul Syst Bio 2008 2 193 201 10.4137/grsb.s702 PMC2733090 19787083

[B37] Erdös P Rényi A On random graphs Publ Math Debrecen 1959 6 290 297

[B38] Barabási A Emergence of Scaling in Random Networks Science 1999 286 509 512 10.1126/science.286.5439.509 10521342

[B39] Burchard J Zhang C Liu AM Poon RT Lee NP Wong KF Sham PC Lam BY Ferguson MD Tokiwa G Smith R Leeson B Beard R Lamb JR Lim L Mao M Dai H Luk JM microRNA-122 as a regulator of mitochondrial metabolic gene network in hepatocellular carcinoma Mol Syst Biol 2010 6 402 20739924 10.1038/msb.2010.58 PMC2950084

[B40] Benjamini Y Hochberg Y Controlling the False Discovery Rate: A Practical and Powerful Approach to Multiple Testing Journal of the Royal Statistical Society. Series B (Methodological) 1995 57 289

[B41] Pellegrino R Calvisi DF Ladu S Ehemann V Staniscia T Evert M Dombrowski F Schirmacher P Longerich T Oncogenic and tumor suppressive roles of polo-like kinases in human hepatocellular carcinoma Hepatology 2010 51 857 868 20112253 10.1002/hep.23467

[B42] Whittaker S Marais R Zhu AX The role of signaling pathways in the development and treatment of hepatocellular carcinoma Oncogene 2010 29 4989 5005 10.1038/onc.2010.236 20639898

[B43] Pearson G Robinson F Beers Gibson T Xu BE Karandikar M Berman K Cobb MH Mitogen-activated protein (MAP) kinase pathways: regulation and physiological functions Endocr Rev 2001 22 153 183 10.1210/er.22.2.153 11294822

[B44] Liu P Kimmoun E Legrand A Sauvanet A Degott C Lardeux B Bernuau D Activation of NF-kappaB, AP-1 and STAT transcription factors is a frequent and early event in human hepatocellular carcinomas J Hepatol 2002 37 63 71 10.1016/S0168-8278(02)00064-8 12076863

[B45] Ribatti D Marzullo A Gentile A Longo V Nico B Vacca A Dammacco F Erythropoietin/erythropoietin-receptor system is involved in angiogenesis in human hepatocellular carcinoma Histopathology 2007 50 591 596 10.1111/j.1365-2559.2007.02654.x 17394495 PMC1891001

[B46] van Dam H Castellazzi M Distinct roles of Jun: Fos and Jun: ATF dimers in oncogenesis Oncogene 2001 20 2453 2464 10.1038/sj.onc.1204239 11402340

[B47] Meng Q Xia Y c-Jun, at the crossroad of the signaling network Protein Cell 2011 2 889 898 10.1007/s13238-011-1113-3 22180088 PMC4875184

[B48] Cross MJ Dixelius J Matsumoto T Claesson-Welsh L VEGF-receptor signal transduction Trends Biochem Sci 2003 28 488 494 10.1016/S0968-0004(03)00193-2 13678960

[B49] Demir E Cary MP Paley S The BioPAX community standard for pathway data sharing Nat Biotechnol 2010 28 935 942 10.1038/nbt.1666 20829833 PMC3001121

[B50] Seal RL Gordon SM Lush MJ Wright MW Bruford EA genenames.org: the HGNC resources in 2011 Nucleic Acids Res 2011 39 D514 D519 10.1093/nar/gkq892 20929869 PMC3013772

[B51] Consortium UniProt The Universal Protein Resource (UniProt) in 2010 Nucleic Acids Res 2010 38 D142 D148 19843607 10.1093/nar/gkp846 PMC2808944

[B52] Tian L Greenberg SA Kong SW Altschuler J Kohane IS Park PJ Discovering statistically significant pathways in expression profiling studies Proc Natl Acad Sci USA 2005 102 13544 13549 10.1073/pnas.0506577102 16174746 PMC1200092

[B53] Mootha VK Lindgren CM Eriksson KF Subramanian A Sihag S Lehar J Puigserver P Carlsson E Ridderstrale M Laurila E Houstis N Daly MJ Patterson N Mesirov JP Golub TR Tamayo P Spiegelman B Lander ES Hirschhorn JN Altshuler D Groop LC PGC-1alpha-responsive genes involved in oxidative phosphorylation are coordinately downregulated in human diabetes Nat Genet 2003 34 267 273 10.1038/ng1180 12808457

[B54] Csardi G Nepusz T The igraph software package for complex network research InterJournal Complex Systems 2006 1695

[B55] Lopes CT Franz M Kazi F Donaldson SL Morris Q Bader GD Cytoscape Web: an interactive web-based network browser Bioinformatics 2010 26 2347 2348 10.1093/bioinformatics/btq430 20656902 PMC2935447

