# Supplementary figures and images for: Centrality-based pathway enrichment: a systematic approach for finding significant pathways dominated by key genes
Source: BMC Syst Biol. 2012 Jun 6;6:56. doi: 10.1186/1752-0509-6-56 (PMC3443660; doi:10.1186/1752-0509-6-56)

ER random network (200 nodes)

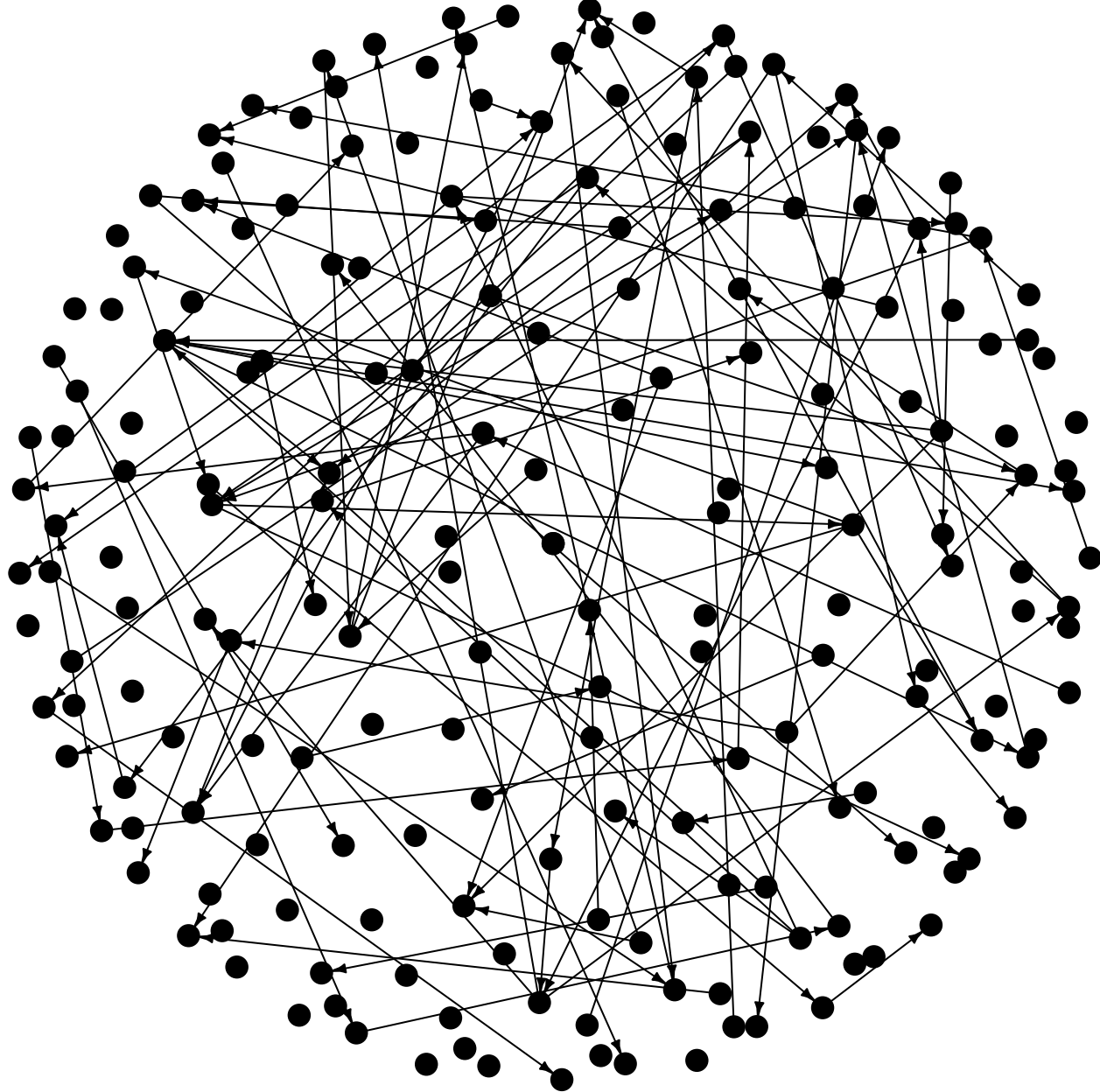

BA random network (200 nodes)

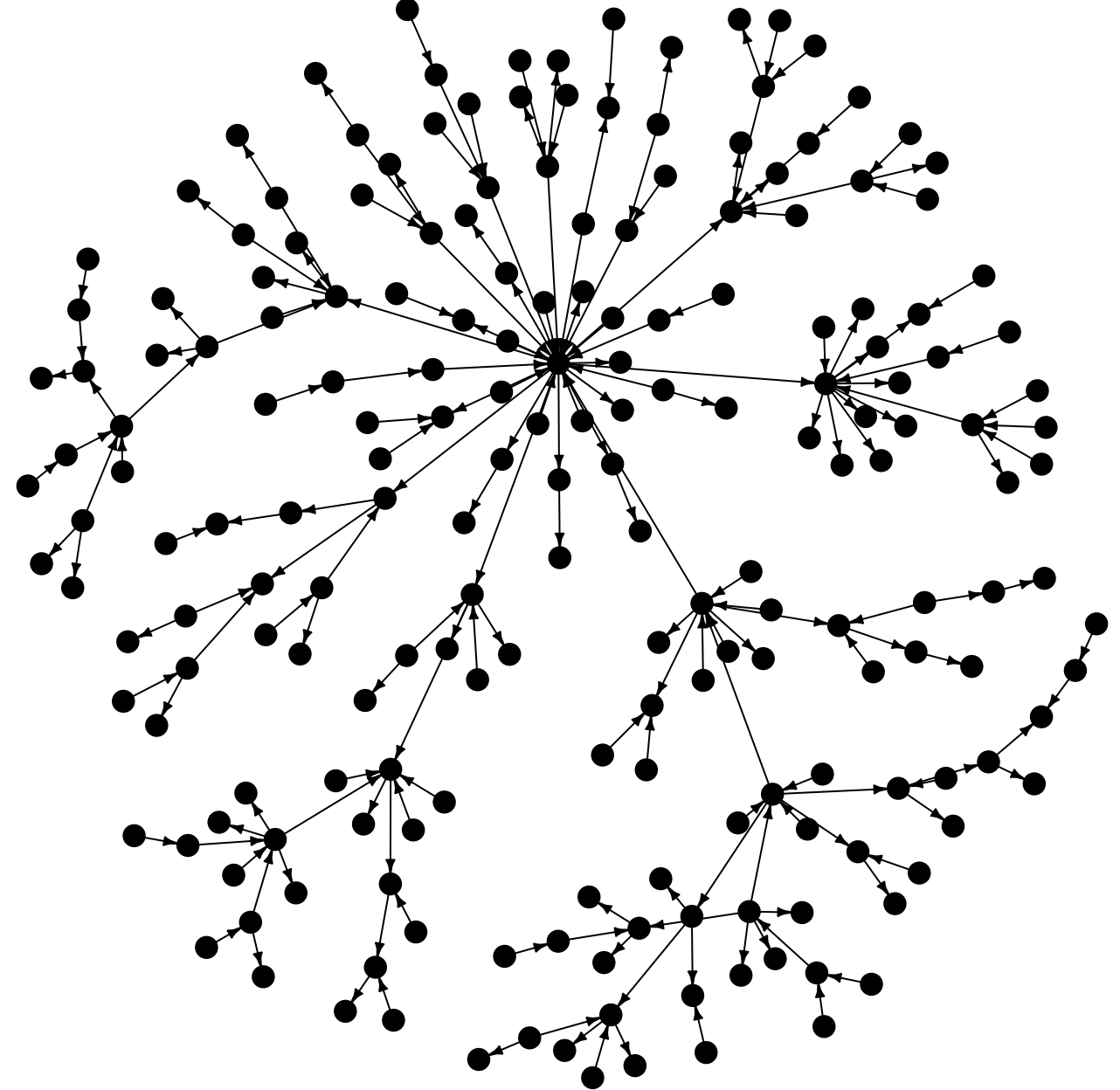

Supplement: Additional file 1 — Two random pathways generated from ER model and BA model. Number of nodes in both networks is 200. [file 1752-0509-6-56-S1.pdf]
