# Supplementary material for: Centrality-based pathway enrichment: a systematic approach for finding significant pathways dominated by key genes
Source: BMC Syst Biol. 2012 Jun 6;6:56. doi: 10.1186/1752-0509-6-56 (PMC3443660; doi:10.1186/1752-0509-6-56)
Supplement: Additional file 2 — Histograms of in-largest reach aggregated from 1000 networks generated either by ER model or BA model. [file 1752-0509-6-56-S2.pdf]

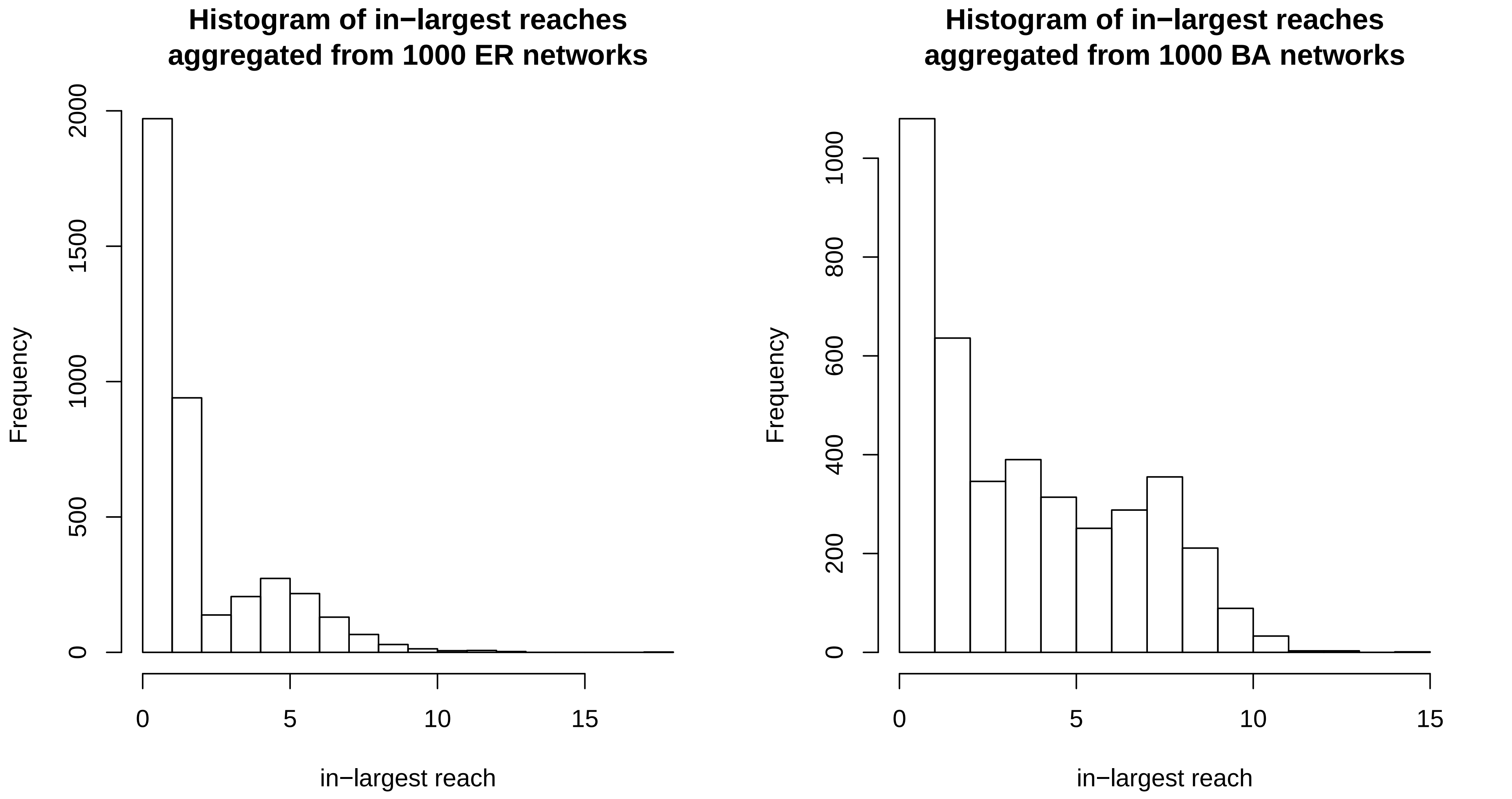
Histogram of in−largest reaches
aggregated from 1000 ER networks
Frequency
0
500
1000
1500
2000
0
5
10
15
in−largest reach
Histogram of in−largest reaches
aggregated from 1000 BA networks
Frequency
0
200
400
600
800
1000
0
5
10
15
in−largest reach
