# Supplementary material for: Centrality-based pathway enrichment: a systematic approach for finding significant pathways dominated by key genes
Source: BMC Syst Biol. 2012 Jun 6;6:56. doi: 10.1186/1752-0509-6-56 (PMC3443660; doi:10.1186/1752-0509-6-56)
Supplement: Additional file 3 — The complete list of p-values of pathways generated under different centrality measurements. [file 1752-0509-6-56-S3.doc]

**Table 1. Complete list of p-values of pathways under different centrality measurements.** The cutoff is set to 0.01.

| **Pathway** | **equal.weight** | **in.degree** | **out.degree** | **betweenness** | **in.reach** | **out.reach** | **ORA** | **Reference** |
| --- | --- | --- | --- | --- | --- | --- | --- | --- |
|  | Evaluated as significant by traditional ORA and CePa | | | | | | | |
| foxm1pathway | 0.000 | 0.000 | 0.000 | 0.000 | 0.000 | 0.000 | 0.000 |  |
| plk1_pathway | 0.000 | 0.000 | 0.000 | 0.001 | 0.000 | 0.000 | 0.000 | [1] |
| aurora_b_pathway | 0.000 | 0.002 | 0.000 | 0.000 | 0.000 | 0.000 | 0.000 |  |
| fanconi_pathway | 0.000 | 0.000 | 0.000 | 0.001 | 0.000 | 0.000 | 0.000 |  |
| aurora_a_pathway | 0.001 | 0.001 | 0.000 | 0.000 | 0.000 | 0.006 | 0.000 |  |
| er_nongenomic_pathway | 0.000 | 0.001 | 0.001 | 0.000 | 0.001 | 0.002 | 0.001 |  |
| pdgfrapathway | 0.007 | 0.006 | 0.002 | 0.005 | 0.003 | 0.000 | 0.005 | [2] |
| ap1_pathway | 0.001 | 0.002 | 0.001 | 0.000 | 0.002 | 0.000 | 0.007 | [3] |
| bard1pathway | 0.000 | 0.000 | 0.000 | 0.000 | 0.016 | 0.001 | 0.000 |  |
| fak_pathway | 0.010 | 0.006 | 0.003 | 0.054 | 0.007 | 0.009 | 0.003 | [4] |
| atr_pathway | 0.000 | 0.000 | 0.001 | 0.027 | 0.000 | 0.038 | 0.000 |  |
| met_pathway | 0.004 | 0.001 | 0.014 | 0.016 | 0.006 | 0.006 | 0.004 | [2] |
| e2f_pathway | 0.002 | 0.000 | 0.211 | 0.111 | 0.000 | 0.165 | 0.000 |  |
| trkrpathway | 0.007 | 0.015 | 0.009 | 0.054 | 0.020 | 0.007 | 0.002 |  |
| mapktrkpathway | 0.002 | 0.003 | 0.093 | 0.160 | 0.001 | 0.081 | 0.002 | [2] |
| il2_1pathway | 0.009 | 0.015 | 0.045 | 0.048 | 0.011 | 0.005 | 0.002 |  |
| endothelinpathway | 0.014 | 0.047 | 0.187 | 0.032 | 0.003 | 0.003 | 0.006 |  |
| erbb2erbb3pathway | 0.007 | 0.009 | 0.019 | 0.031 | 0.014 | 0.021 | 0.009 | [5] |
|  | Evaluated as significant by traditional ORA only | | | | | | | |
| lysophospholipid_pathway | 0.046 | 0.034 | 0.196 | 0.231 | 0.021 | 0.170 | 0.001 |  |
| ar_nongenomic_pathway | 0.035 | 0.032 | 0.136 | 0.206 | 0.020 | 0.099 | 0.001 |  |
| fcer1pathway | 0.079 | 0.067 | 0.406 | 0.196 | 0.044 | 0.026 | 0.001 |  |
| ar_tf_pathway | 0.153 | 0.525 | 0.185 | 0.661 | 0.528 | 0.218 | 0.003 |  |
| atf2_pathway | 0.074 | 0.102 | 0.111 | 0.111 | 0.076 | 0.078 | 0.003 |  |
| cmyb_pathway | 0.135 | 0.365 | 0.527 | 0.855 | 0.114 | 0.527 | 0.008 |  |
| p73pathway | 0.127 | 0.268 | 0.224 | 0.379 | 0.184 | 0.071 | 0.008 | [6] |
| nfat_tfpathway | 0.063 | 0.301 | 0.157 | 0.324 | 0.268 | 0.011 | 0.009 |  |
|  | Evaluated as significant by CePa only | | | | | | | |
| vegfr1_2_pathway | 0.006 | 0.002 | 0.007 | 0.008 | 0.002 | 0.048 | 0.171 | [2] |
| plk3_pathway | 0.006 | 0.013 | 0.004 | 0.007 | 0.009 | 0.012 | 0.011 | [1] |
| il12_stat4pathway | 0.013 | 0.114 | 0.110 | 0.001 | 0.002 | 0.003 | 0.098 |  |
| txa2pathway | 0.014 | 0.001 | 0.007 | 0.040 | 0.031 | 0.007 | 0.240 |  |
| epha2_fwdpathway | 0.003 | 0.023 | 0.026 | 0.022 | 0.011 | 0.010 | 0.012 | [7] |
| syndecan_3_pathway | 0.029 | 0.040 | 0.009 | 0.026 | 0.043 | 0.005 | 0.021 |  |
| cxcr4_pathway | 0.011 | 0.007 | 0.009 | 0.039 | 0.013 | 0.014 | 0.056 | [8] |
| epopathway | 0.070 | 0.025 | 0.010 | 0.002 | 0.065 | 0.024 | 0.098 | [9] |
| toll_endogenous_pathway | 0.051 | 0.072 | 0.004 | 0.026 | 0.050 | 0.014 | 0.014 | [10] |
| epha_fwdpathway | 0.040 | 0.010 | 0.014 | 0.061 | 0.045 | 0.070 | 0.022 | [7] |
| atm_pathway | 0.112 | 0.203 | 0.305 | 0.503 | 0.009 | 0.540 | 0.026 |  |
| angiopoietinreceptor_pathway | 0.017 | 0.077 | 0.010 | 0.058 | 0.043 | 0.013 | 0.034 |  |
| anthraxpathway | 0.016 | 0.004 | 0.022 | 0.018 | 0.016 | 0.040 | 0.047 |  |
| il1pathway | 0.031 | 0.022 | 0.025 | 0.037 | 0.147 | 0.000 | 0.068 |  |
| thrombin_par1_pathway | 0.123 | 0.024 | 0.069 | 0.004 | 0.024 | 0.069 | 0.087 |  |
| alk1pathway | 0.061 | 0.032 | 0.037 | 0.018 | 0.010 | 0.046 | 0.143 |  |
| p75ntrpathway | 0.026 | 0.007 | 0.015 | 0.041 | 0.062 | 0.085 | 0.196 | [11] |
| alphasynuclein_pathway | 0.064 | 0.035 | 0.083 | 0.008 | 0.023 | 0.065 | 0.460 |  |
|  | Evaluated as insignificant both by traditional ORA and by CePa | | | | | | | |
| il12_2pathway | 0.155 | 0.454 | 0.528 | 0.538 | 0.228 | 0.081 | 0.011 |  |
| s1p_s1p1_pathway | 0.074 | 0.140 | 0.161 | 0.139 | 0.051 | 0.044 | 0.012 |  |
| p53downstreampathway | 0.119 | 0.327 | 0.380 | 0.384 | 0.066 | 0.299 | 0.013 |  |
| fra_pathway | 0.035 | 0.033 | 0.101 | 0.125 | 0.017 | 0.062 | 0.015 |  |
| rb_1pathway | 0.131 | 0.300 | 0.157 | 0.394 | 0.307 | 0.025 | 0.018 |  |
| hdac_classii_pathway | 0.044 | 0.121 | 0.121 | 0.296 | 0.112 | 0.184 | 0.026 |  |
| il6_7pathway | 0.044 | 0.067 | 0.089 | 0.117 | 0.041 | 0.053 | 0.026 |  |
| hedgehog_glipathway | 0.250 | 0.476 | 0.128 | 0.420 | 0.604 | 0.033 | 0.030 |  |
| gmcsf_pathway | 0.403 | 0.378 | 0.427 | 0.378 | 0.229 | 0.408 | 0.035 |  |
| cd8tcrdownstreampathway | 0.024 | 0.163 | 0.084 | 0.089 | 0.142 | 0.063 | 0.036 |  |
| s1p_s1p2_pathway | 0.163 | 0.176 | 0.072 | 0.100 | 0.138 | 0.230 | 0.039 |  |
| erbb4_pathway | 0.022 | 0.055 | 0.018 | 0.043 | 0.044 | 0.020 | 0.044 |  |
| tcrcalciumpathway | 0.089 | 0.324 | 0.056 | 0.310 | 0.344 | 0.135 | 0.046 |  |
| s1p_s1p5_pathway | 0.146 | 0.205 | 0.146 | 0.060 | 0.065 | 0.146 | 0.047 |  |
| ptp1bpathway | 0.251 | 0.294 | 0.294 | 0.530 | 0.351 | 0.357 | 0.049 |  |
| fgf_pathway | 0.054 | 0.111 | 0.122 | 0.627 | 0.230 | 0.059 | 0.055 |  |
| reg_gr_pathway | 0.445 | 0.840 | 0.260 | 0.427 | 0.343 | 0.358 | 0.056 |  |
| tcrjnkpathway | 0.029 | 0.017 | 0.035 | 0.036 | 0.017 | 0.040 | 0.056 |  |
| tcrraspathway | 0.323 | 0.260 | 0.295 | 0.253 | 0.217 | 0.310 | 0.056 |  |
| s1p_s1p4_pathway | 0.316 | 0.306 | 0.302 | 0.298 | 0.223 | 0.314 | 0.056 |  |
| wnt_canonical_pathway | 0.304 | 0.423 | 0.325 | 0.371 | 0.391 | 0.231 | 0.057 |  |
| ephbfwdpathway | 0.214 | 0.198 | 0.212 | 0.247 | 0.089 | 0.215 | 0.061 |  |
| deltanp63pathway | 0.103 | 0.111 | 0.148 | 0.131 | 0.033 | 0.577 | 0.062 |  |
| et_egfrpathway | 0.121 | 0.212 | 0.128 | 0.221 | 0.032 | 0.217 | 0.064 |  |
| hes_heypathway | 0.398 | 0.557 | 0.724 | 0.691 | 0.300 | 0.808 | 0.069 |  |
| nephrin_neph1_pathway | 0.417 | 0.430 | 0.384 | 0.481 | 0.255 | 0.341 | 0.075 |  |
| pi3kplctrkpathway | 0.474 | 0.559 | 0.192 | 0.676 | 0.821 | 0.158 | 0.087 |  |
| prlsignalingeventspathway | 0.130 | 0.096 | 0.259 | 0.135 | 0.039 | 0.134 | 0.095 |  |
| pi3kcipathway | 0.256 | 0.395 | 0.449 | 0.569 | 0.212 | 0.232 | 0.097 |  |
| telomerasepathway | 0.127 | 0.201 | 0.190 | 0.171 | 0.287 | 0.100 | 0.104 |  |
| integrin4_pathway | 0.052 | 0.052 | 0.023 | 1.000 | 0.059 | 0.053 | 0.108 |  |
| erbb1_internalization_pathway | 0.244 | 0.251 | 0.229 | 0.234 | 0.256 | 0.455 | 0.110 |  |
| tcptp_pathway | 0.259 | 0.438 | 0.392 | 0.535 | 0.193 | 0.095 | 0.110 |  |
| syndecan_4_pathway | 0.202 | 0.198 | 0.230 | 0.226 | 0.176 | 0.294 | 0.111 |  |
| ret_pathway | 0.364 | 0.387 | 0.383 | 0.517 | 0.352 | 0.426 | 0.122 |  |
| erbb1_receptor_proximal_pathway | 0.269 | 0.244 | 0.251 | 0.338 | 0.289 | 0.323 | 0.125 |  |
| netrin_pathway | 0.246 | 0.238 | 0.240 | 0.240 | 0.256 | 0.227 | 0.125 |  |
| glypican_3pathway | 0.054 | 0.056 | 0.046 | 0.019 | 0.025 | 0.054 | 0.131 |  |
| pdgf_pathway | 0.101 | 0.050 | 0.065 | 1.000 | 0.061 | 0.097 | 0.131 |  |
| cxcr3pathway | 0.369 | 0.230 | 0.229 | 0.020 | 0.218 | 0.175 | 0.135 |  |
| arf_3pathway | 0.163 | 0.149 | 0.169 | 0.145 | 0.164 | 0.167 | 0.142 |  |
| erbb1_downstream_pathway | 0.181 | 0.159 | 0.417 | 0.210 | 0.041 | 0.155 | 0.143 |  |
| era_genomic_pathway | 0.029 | 0.023 | 0.037 | 0.019 | 0.025 | 0.027 | 0.148 |  |
| p38_mkk3_6pathway | 0.264 | 0.116 | 0.274 | 0.122 | 0.180 | 0.259 | 0.161 |  |
| rapid_gr_pathway | 0.143 | 0.106 | 0.115 | 0.098 | 0.120 | 0.132 | 0.170 |  |
| s1p_meta_pathway | 0.253 | 0.207 | 0.127 | 0.249 | 0.241 | 0.133 | 0.186 |  |
| tgfbrpathway | 0.346 | 0.337 | 0.394 | 0.367 | 0.357 | 0.390 | 0.195 |  |
| igf1_pathway | 0.089 | 0.069 | 0.183 | 0.234 | 0.067 | 0.056 | 0.199 |  |
| lis1pathway | 0.285 | 0.239 | 0.307 | 0.149 | 0.079 | 0.213 | 0.199 |  |
| amb2_neutrophils_pathway | 0.266 | 0.278 | 0.272 | 0.235 | 0.283 | 0.263 | 0.199 |  |
| glypican_2pathway | 0.200 | 0.194 | 0.191 | 1.000 | 0.182 | 0.195 | 0.205 |  |
| il23pathway | 0.601 | 0.710 | 0.742 | 0.827 | 0.705 | 0.559 | 0.205 |  |
| myc_activpathway | 0.373 | 0.393 | 0.406 | 0.537 | 0.351 | 0.590 | 0.206 |  |
| smad2_3nuclearpathway | 0.462 | 0.373 | 0.526 | 0.384 | 0.333 | 0.533 | 0.206 |  |
| hedgehog_2pathway | 0.201 | 0.159 | 0.238 | 0.224 | 0.176 | 0.357 | 0.209 |  |
| cd8tcrpathway | 0.414 | 0.434 | 0.690 | 0.544 | 0.423 | 0.437 | 0.209 |  |
| a4b7_pathway | 0.397 | 0.485 | 0.265 | 0.409 | 0.438 | 0.219 | 0.211 |  |
| erbb_network_pathway | 0.064 | 0.093 | 0.049 | 0.140 | 0.107 | 0.037 | 0.216 |  |
| thrombin_par4_pathway | 0.140 | 0.031 | 0.075 | 0.245 | 0.055 | 0.200 | 0.216 |  |
| p38alphabetapathway | 0.445 | 0.694 | 0.540 | 0.685 | 0.421 | 0.471 | 0.219 |  |
| ephrinbrevpathway | 0.462 | 0.449 | 0.322 | 0.315 | 0.453 | 0.206 | 0.219 |  |
| cdc42_pathway | 0.469 | 0.512 | 0.698 | 0.706 | 0.216 | 0.724 | 0.221 |  |
| rac1_pathway | 0.377 | 0.411 | 0.325 | 0.148 | 0.329 | 0.217 | 0.224 |  |
| betacatenin_nuc_pathway | 0.646 | 0.791 | 0.675 | 0.813 | 0.725 | 0.584 | 0.244 |  |
| bcr_5pathway | 0.710 | 0.695 | 0.743 | 0.812 | 0.492 | 0.799 | 0.252 |  |
| wnt_noncanonical_pathway | 0.104 | 0.027 | 0.123 | 0.052 | 0.075 | 0.293 | 0.260 |  |
| tcr_pathway | 0.142 | 0.149 | 0.154 | 0.158 | 0.220 | 0.104 | 0.266 |  |
| foxopathway | 0.125 | 0.189 | 0.286 | 0.155 | 0.087 | 0.319 | 0.276 |  |
| ecadherin_stabilization_pathway | 0.133 | 0.060 | 0.346 | 0.015 | 0.037 | 0.096 | 0.279 |  |
| myc_pathway | 0.260 | 0.231 | 0.259 | 0.263 | 0.250 | 0.297 | 0.282 |  |
| trail_pathway | 0.364 | 0.493 | 0.366 | 0.548 | 0.425 | 0.330 | 0.282 |  |
| lymphangiogenesis_pathway | 0.660 | 0.709 | 0.616 | 0.519 | 0.470 | 0.408 | 0.282 |  |
| s1p_s1p3_pathway | 0.254 | 0.226 | 0.244 | 0.140 | 0.253 | 0.207 | 0.282 |  |
| aurora_c_pathway | 0.294 | 0.316 | 0.282 | 0.304 | 0.323 | 0.295 | 0.290 |  |
| ephrinarevpathway | 0.277 | 0.304 | 0.301 | 0.288 | 0.139 | 0.310 | 0.290 |  |
| nfat_3pathway | 0.533 | 0.566 | 0.553 | 0.648 | 0.456 | 0.368 | 0.293 |  |
| vegf_vegfr_pathway | 0.166 | 0.082 | 0.060 | 1.000 | 0.084 | 0.293 | 0.295 |  |
| p38gammadeltapathway | 0.364 | 0.311 | 0.361 | 0.455 | 0.310 | 0.249 | 0.295 |  |
| p53regulationpathway | 0.487 | 0.701 | 0.585 | 0.852 | 0.695 | 0.601 | 0.304 |  |
| hif2pathway | 0.537 | 0.678 | 0.405 | 0.569 | 0.645 | 0.303 | 0.304 |  |
| il27pathway | 0.331 | 0.369 | 0.284 | 0.348 | 0.335 | 0.171 | 0.308 |  |
| glypican_1pathway | 0.443 | 0.419 | 0.472 | 0.584 | 0.389 | 0.612 | 0.308 |  |
| ps1pathway | 0.696 | 0.612 | 0.506 | 0.629 | 0.538 | 0.646 | 0.319 |  |
| p38alphabetadownstreampathway | 0.471 | 0.426 | 0.547 | 0.508 | 0.185 | 0.475 | 0.326 |  |
| alk2pathway | 0.166 | 0.164 | 0.197 | 0.133 | 0.147 | 0.179 | 0.337 |  |
| a6b1_a6b4_integrin_pathway | 0.039 | 0.017 | 0.051 | 0.056 | 0.029 | 0.087 | 0.359 |  |
| ilk_pathway | 0.580 | 0.618 | 0.655 | 0.608 | 0.441 | 0.418 | 0.359 |  |
| rhoa_pathway | 0.415 | 0.421 | 0.294 | 0.590 | 0.239 | 0.244 | 0.359 |  |
| il8cxcr1_pathway | 0.180 | 0.202 | 0.184 | 0.279 | 0.242 | 0.315 | 0.359 |  |
| tap63pathway | 0.183 | 0.207 | 0.136 | 0.131 | 0.189 | 0.170 | 0.366 |  |
| syndecan_pathway | 0.359 | 0.349 | 0.373 | 1.000 | 0.368 | 0.346 | 0.367 |  |
| myc_represspathway | 0.583 | 0.583 | 0.585 | 0.689 | 0.447 | 0.509 | 0.371 |  |
| pdgfrbpathway | 0.575 | 0.517 | 0.438 | 0.177 | 0.463 | 0.372 | 0.375 |  |
| reelinpathway | 0.357 | 0.331 | 0.410 | 0.456 | 0.332 | 0.382 | 0.384 |  |
| il4_2pathway | 0.571 | 0.685 | 0.697 | 0.712 | 0.598 | 0.609 | 0.388 |  |
| ecadherin_keratinocyte_pathway | 0.501 | 0.503 | 0.478 | 0.477 | 0.392 | 0.191 | 0.400 |  |
| il2_stat5pathway | 0.800 | 0.718 | 0.758 | 1.000 | 0.724 | 0.773 | 0.410 |  |
| dnapk_pathway | 0.368 | 0.372 | 0.394 | 0.392 | 0.344 | 0.392 | 0.418 |  |
| ajdiss_2pathway | 0.679 | 0.649 | 0.812 | 0.780 | 0.552 | 0.537 | 0.419 |  |
| hif1_tfpathway | 0.755 | 0.793 | 0.797 | 0.791 | 0.644 | 0.827 | 0.422 |  |
| pi3kcibpathway | 0.423 | 0.432 | 0.356 | 0.386 | 0.454 | 0.366 | 0.436 |  |
| ifngpathway | 0.811 | 0.813 | 0.853 | 0.727 | 0.687 | 0.654 | 0.438 |  |
| erb_genomic_pathway | 0.783 | 0.569 | 0.802 | 0.810 | 0.786 | 0.814 | 0.456 |  |
| lkb1_pathway | 0.607 | 0.417 | 0.672 | 0.495 | 0.365 | 0.695 | 0.481 |  |
| integrin_a4b1_pathway | 0.600 | 0.630 | 0.636 | 0.618 | 0.603 | 0.701 | 0.485 |  |
| lpa4_pathway | 0.815 | 0.818 | 0.800 | 0.731 | 0.777 | 0.746 | 0.493 |  |
| arf6downstreampathway | 0.353 | 0.265 | 0.270 | 0.188 | 0.201 | 0.241 | 0.493 |  |
| ar_pathway | 0.491 | 0.422 | 0.540 | 0.719 | 0.592 | 0.579 | 0.494 |  |
| hdac_classi_pathway | 0.413 | 0.307 | 0.446 | 0.374 | 0.276 | 0.576 | 0.498 |  |
| kitpathway | 0.336 | 0.097 | 0.255 | 0.320 | 0.352 | 0.114 | 0.498 |  |
| hnf3apathway | 0.712 | 0.960 | 0.145 | 0.749 | 0.965 | 0.153 | 0.503 |  |
| cd40_pathway | 0.294 | 0.212 | 0.264 | 0.337 | 0.184 | 0.274 | 0.509 |  |
| ncadherinpathway | 0.656 | 0.628 | 0.642 | 0.579 | 0.337 | 0.358 | 0.509 |  |
| il8cxcr2_pathway | 0.280 | 0.254 | 0.237 | 0.330 | 0.379 | 0.226 | 0.509 |  |
| insulin_pathway | 0.339 | 0.286 | 0.271 | 0.353 | 0.376 | 0.242 | 0.524 |  |
| ceramide_pathway | 0.719 | 0.489 | 0.464 | 0.207 | 0.610 | 0.449 | 0.524 |  |
| circadianpathway | 0.458 | 0.460 | 0.435 | 0.543 | 0.583 | 0.406 | 0.529 |  |
| arf6_pathway | 0.804 | 0.870 | 0.711 | 0.871 | 0.876 | 0.752 | 0.532 |  |
| il2_pi3kpathway | 0.824 | 0.863 | 0.619 | 0.516 | 0.695 | 0.635 | 0.532 |  |
| vegfr1_pathway | 0.372 | 0.291 | 0.240 | 0.060 | 0.332 | 0.268 | 0.544 |  |
| hdac_classiii_pathway | 0.460 | 0.374 | 0.483 | 0.596 | 0.553 | 0.564 | 0.544 |  |
| rxr_vdr_pathway | 0.448 | 0.425 | 0.433 | 0.457 | 0.418 | 0.417 | 0.544 |  |
| ecadherin_nascentaj_pathway | 0.745 | 0.804 | 0.723 | 0.901 | 0.762 | 0.766 | 0.555 |  |
| smad2_3pathway | 0.656 | 0.668 | 0.335 | 0.631 | 0.625 | 0.564 | 0.562 |  |
| integrin1_pathway | 0.229 | 0.232 | 0.170 | 0.516 | 0.201 | 0.303 | 0.580 |  |
| betacatenin_deg_pathway | 0.787 | 0.781 | 0.790 | 0.786 | 0.759 | 0.740 | 0.594 |  |
| rac1_reg_pathway | 0.675 | 0.667 | 0.611 | 0.679 | 0.683 | 0.597 | 0.600 |  |
| notch_pathway | 0.398 | 0.259 | 0.324 | 0.246 | 0.262 | 0.287 | 0.608 |  |
| integrin2_pathway | 0.761 | 0.755 | 0.754 | 0.775 | 0.775 | 0.693 | 0.620 |  |
| hif1apathway | 0.636 | 0.729 | 0.653 | 0.663 | 0.629 | 0.503 | 0.624 |  |
| retinoic_acid_pathway | 0.273 | 0.272 | 0.339 | 0.456 | 0.285 | 0.276 | 0.644 |  |
| avb3_opn_pathway | 0.777 | 0.570 | 0.614 | 0.326 | 0.693 | 0.579 | 0.666 |  |
| p38_mk2pathway | 0.766 | 0.540 | 0.752 | 0.282 | 0.662 | 0.762 | 0.679 |  |
| bmppathway | 0.540 | 0.738 | 0.709 | 0.671 | 0.449 | 0.606 | 0.679 |  |
| integrin3_pathway | 0.773 | 0.727 | 0.758 | 0.774 | 0.766 | 0.758 | 0.697 |  |
| hnf3bpathway | 0.769 | 0.679 | 0.793 | 0.763 | 0.640 | 0.714 | 0.715 |  |
| ranbp2pathway | 0.453 | 0.296 | 0.451 | 0.313 | 0.375 | 0.410 | 0.716 |  |
| cone_pathway | 0.832 | 0.847 | 0.805 | 0.850 | 0.846 | 0.541 | 0.727 |  |
| rhodopsin_pathway | 0.862 | 0.849 | 0.834 | 0.859 | 0.844 | 0.632 | 0.727 |  |
| rhoa_reg_pathway | 0.188 | 0.086 | 0.094 | 0.089 | 0.102 | 0.493 | 0.731 |  |
| hivnefpathway | 0.706 | 0.626 | 0.522 | 0.680 | 0.896 | 0.496 | 0.745 |  |
| tnfpathway | 0.328 | 0.250 | 0.292 | 0.156 | 0.292 | 0.159 | 0.747 |  |
| arf6_traffickingpathway | 0.892 | 0.862 | 0.933 | 0.915 | 0.870 | 0.913 | 0.747 |  |
| syndecan_2_pathway | 0.842 | 0.874 | 0.387 | 0.888 | 0.914 | 0.378 | 0.762 |  |
| mtor_4pathway | 0.798 | 0.753 | 0.633 | 0.565 | 0.821 | 0.402 | 0.770 |  |
| il3_pathway | 0.564 | 0.444 | 0.583 | 0.617 | 0.481 | 0.715 | 0.788 |  |
| syndecan_1_pathway | 0.573 | 0.339 | 0.457 | 0.180 | 0.483 | 0.404 | 0.794 |  |
| avb3_integrin_pathway | 0.364 | 0.233 | 0.476 | 0.239 | 0.299 | 0.416 | 0.814 |  |
| caspase_pathway | 0.553 | 0.461 | 0.509 | 0.634 | 0.484 | 0.554 | 0.816 |  |
| upa_upar_pathway | 0.882 | 0.876 | 0.896 | 0.770 | 0.829 | 0.932 | 0.847 |  |
| cdc42_reg_pathway | 0.870 | 0.837 | 0.849 | 0.859 | 0.854 | 0.844 | 0.850 |  |
| nectin_pathway | 0.890 | 0.868 | 0.854 | 0.515 | 0.828 | 0.795 | 0.850 |  |
| integrin5_pathway | 0.850 | 0.855 | 0.842 | 0.842 | 0.868 | 0.837 | 0.857 |  |
| faspathway | 0.810 | 0.818 | 0.854 | 0.802 | 0.602 | 0.946 | 0.905 |  |
| pi3kciaktpathway | 0.835 | 0.841 | 0.804 | 0.608 | 0.748 | 0.547 | 0.905 |  |
| nfkappabcanonicalpathway | 0.924 | 0.922 | 0.932 | 0.945 | 0.940 | 0.917 | 0.928 |  |
| integrin_a9b1_pathway | 0.205 | 0.204 | 0.205 | 0.242 | 0.220 | 0.270 | 0.943 |  |
| wnt_signaling_pathway | 0.895 | 0.880 | 0.904 | 0.882 | 0.790 | 0.918 | 0.959 |  |
| ephrina_ephapathway | 1.000 | 1.000 | 1.000 | 1.000 | 1.000 | 1.000 | 1.000 |  |
| glypicanpathway | 1.000 | 1.000 | 1.000 | 1.000 | 1.000 | 1.000 | 1.000 |  |
| hnf3pathway | 1.000 | 1.000 | 1.000 | 1.000 | 1.000 | 1.000 | 1.000 |  |
| ephrinb_ephbpathway | 1.000 | 1.000 | 1.000 | 1.000 | 1.000 | 1.000 | 1.000 |  |
| integrin_cs_pathway | 1.000 | 1.000 | 1.000 | 1.000 | 1.000 | 1.000 | 1.000 |  |
| aurora_kinase_pathway | 1.000 | 1.000 | 1.000 | 1.000 | 1.000 | 1.000 | 1.000 |  |
| plk_pathway | 1.000 | 1.000 | 1.000 | 1.000 | 1.000 | 1.000 | 1.000 |  |
| p63pathway | 1.000 | 1.000 | 1.000 | 1.000 | 1.000 | 1.000 | 1.000 |  |
| thrombin_pathway | 1.000 | 1.000 | 1.000 | 1.000 | 1.000 | 1.000 | 1.000 |  |
| gr_signal_pathway | 1.000 | 1.000 | 1.000 | 1.000 | 1.000 | 1.000 | 1.000 |  |
| insulin_glucose_pathway | 1.000 | 1.000 | 1.000 | 1.000 | 1.000 | 1.000 | 1.000 |  |
| alk1_2pathway | 1.000 | 1.000 | 1.000 | 1.000 | 1.000 | 1.000 | 1.000 |  |
| il8_pathway | 1.000 | 1.000 | 1.000 | 1.000 | 1.000 | 1.000 | 1.000 |  |
| nfkappabalternativepathway | 1.000 | 1.000 | 1.000 | 1.000 | 1.000 | 1.000 | 1.000 |  |
| ecadherin_1_pathway | 1.000 | 1.000 | 1.000 | 1.000 | 1.000 | 1.000 | 1.000 |  |
| androgen_pathway | 1.000 | 1.000 | 1.000 | 1.000 | 1.000 | 1.000 | 1.000 |  |
| il5_pathway | 1.000 | 1.000 | 1.000 | 1.000 | 1.000 | 1.000 | 1.000 |  |
| integrin7_pathway | 1.000 | 1.000 | 1.000 | 1.000 | 1.000 | 1.000 | 1.000 |  |
| nfkappabatypicalpathway | 1.000 | 1.000 | 1.000 | 1.000 | 1.000 | 1.000 | 1.000 |  |
| botulinumtoxinpathway | 1.000 | 1.000 | 1.000 | 1.000 | 1.000 | 1.000 | 1.000 |  |
| plk2_4pathway | 1.000 | 1.000 | 1.000 | 1.000 | 1.000 | 1.000 | 1.000 |  |

**Reference:**

1. Pellegrino R, Calvisi DF, Ladu S, et al.: **Oncogenic and tumor suppressive roles of polo-like kinases in human hepatocellular carcinoma.** *Hepatology (Baltimore, Md.)* 2010, **51**:857-68.

2. Whittaker S, Marais R, Zhu AX: **The role of signaling pathways in the development and treatment of hepatocellular carcinoma.** *Oncogene* 2010, **29**:4989-5005.

3. Liu P: **Activation of NF-kappaB, AP-1 and STAT transcription factors is a frequent and early event in human hepatocellular carcinomas**. *Journal of Hepatology* 2002, **37**:63-71.

4. Itoh S: **Role of Expression of Focal Adhesion Kinase in Progression of Hepatocellular Carcinoma**. *Clinical Cancer Research* 2004, **10**:2812-2817.

5. Berasain C, Castillo J, Prieto J, Avila MA: **New molecular targets for hepatocellular carcinoma: the ErbB1 signaling system.** *Liver international : official journal of the International Association for the Study of the Liver* 2007, **27**:174-85.

6. Tannapfel A, Wasner M, Krause K, et al.: **Expression of p73 and Its Relation to Histopathology and Prognosis in Hepatocellular Carcinoma**. *JNCI Journal of the National Cancer Institute* 1999, **91**:1154-1158.

7. Yang P, Yuan W, He J, et al.: **Overexpression of EphA2, MMP-9, and MVD-CD34 in hepatocellular carcinoma: Implications for tumor progression and prognosis.** *Hepatology research : the official journal of the Japan Society of Hepatology* 2009, **39**:1169-77.

8. Shibuta K, Mori M, Shimoda K, et al.: **Regional Expression of CXCL12/CXCR4 in Liver and Hepatocellular Carcinoma and Cell-cycle Variation during in vitro Differentiation**. *Cancer Science* 2002, **93**:789-797.

9. Ribatti D, Marzullo A, Gentile A, et al.: **Erythropoietin/erythropoietin-receptor system is involved in angiogenesis in human hepatocellular carcinoma.** *Histopathology* 2007, **50**:591-6.

10. Maeda S: **NF-κB, JNK, and TLR Signaling Pathways in Hepatocarcinogenesis.** *Gastroenterology research and practice* 2010, **2010**:367694.

11. Tokusashi Y, Asai K, Tamakawa S, et al.: **Expression of NGF in hepatocellular carcinoma cells with its receptors in non-tumor cell components.** *International journal of cancer. Journal international du cancer* 2005, **114**:39-45.
