# Supplementary material for: Centrality-based pathway enrichment: a systematic approach for finding significant pathways dominated by key genes
Source: BMC Syst Biol. 2012 Jun 6;6:56. doi: 10.1186/1752-0509-6-56 (PMC3443660; doi:10.1186/1752-0509-6-56)
Supplement: Additional file 4 — The complete list of FDRs of pathways generated under different centrality measurements. [file 1752-0509-6-56-S4.doc]

**Table 1. Complete list of FDRs of pathways under different centrality measurements.** FDR is calculated by BH process and the cutoff is set to 0.05.

| **pathway** | **equal.weight** | **in.degree** | **out.degree** | **betweenness** | **in.reach** | **out.reach** | **ORA** | **Reference** |
| --- | --- | --- | --- | --- | --- | --- | --- | --- |
|  | Evaluated as significant by traditional ORA and CePa | | | | | | | |
| aurora_b_pathway | 0.000 | 0.034 | 0.000 | 0.000 | 0.000 | 0.000 | 0.000 |  |
| plk1_pathway | 0.000 | 0.000 | 0.000 | 0.025 | 0.000 | 0.000 | 0.000 | [1] |
| foxm1pathway | 0.000 | 0.000 | 0.000 | 0.000 | 0.000 | 0.000 | 0.000 |  |
| fanconi_pathway | 0.000 | 0.000 | 0.000 | 0.025 | 0.000 | 0.000 | 0.000 |  |
| er_nongenomic_pathway | 0.000 | 0.022 | 0.025 | 0.000 | 0.025 | 0.050 | 0.019 |  |
| bard1pathway | 0.000 | 0.000 | 0.000 | 0.000 | 0.140 | 0.028 | 0.001 |  |
| aurora_a_pathway | 0.025 | 0.022 | 0.000 | 0.000 | 0.000 | 0.089 | 0.007 |  |
| atr_pathway | 0.000 | 0.000 | 0.025 | 0.232 | 0.000 | 0.255 | 0.000 |  |
| e2f_pathway | 0.041 | 0.000 | 0.591 | 0.505 | 0.000 | 0.508 | 0.000 |  |
| mapktrkpathway | 0.041 | 0.048 | 0.446 | 0.541 | 0.025 | 0.347 | 0.028 | [2] |
| met_pathway | 0.069 | 0.022 | 0.142 | 0.202 | 0.089 | 0.089 | 0.048 | [2] |
|  | Evaluated as significant by traditional ORA only | | | | | | | |
| lysophospholipid_pathway | 0.256 | 0.205 | 0.571 | 0.631 | 0.156 | 0.508 | 0.023 |  |
| ar_nongenomic_pathway | 0.223 | 0.204 | 0.514 | 0.631 | 0.154 | 0.378 | 0.023 |  |
| fcer1pathway | 0.339 | 0.318 | 0.686 | 0.616 | 0.213 | 0.200 | 0.027 |  |
| trkrpathway | 0.087 | 0.139 | 0.112 | 0.316 | 0.154 | 0.092 | 0.028 |  |
| il2_1pathway | 0.106 | 0.139 | 0.302 | 0.306 | 0.117 | 0.086 | 0.028 |  |
| fak_pathway | 0.112 | 0.079 | 0.061 | 0.316 | 0.098 | 0.112 | 0.039 | [3] |
| ar_tf_pathway | 0.467 | 0.775 | 0.571 | 0.928 | 0.755 | 0.549 | 0.039 |  |
| atf2_pathway | 0.324 | 0.406 | 0.495 | 0.505 | 0.287 | 0.347 | 0.043 |  |
|  | Evaluated as significant by CePa only | | | | | | | |
| ap1_pathway | 0.025 | 0.034 | 0.025 | 0.000 | 0.037 | 0.000 | 0.071 | [4] |
| pdgfrapathway | 0.087 | 0.079 | 0.045 | 0.093 | 0.048 | 0.000 | 0.056 | [2] |
| il12_stat4pathway | 0.130 | 0.424 | 0.495 | 0.025 | 0.037 | 0.061 | 0.322 |  |
| vegfr1_2_pathway | 0.087 | 0.034 | 0.104 | 0.119 | 0.037 | 0.282 | 0.444 | [2] |
| endothelinpathway | 0.130 | 0.262 | 0.571 | 0.255 | 0.048 | 0.061 | 0.069 |  |
| il1pathway | 0.209 | 0.177 | 0.199 | 0.275 | 0.449 | 0.000 | 0.252 |  |
| epopathway | 0.319 | 0.180 | 0.112 | 0.045 | 0.262 | 0.198 | 0.322 | [5] |
| txa2pathway | 0.130 | 0.022 | 0.104 | 0.277 | 0.192 | 0.092 | 0.504 |  |
|  | Evaluated as insignificant both by traditional ORA and by CePa | | | | | | | |
| erbb2erbb3pathway | 0.087 | 0.100 | 0.169 | 0.255 | 0.136 | 0.180 | 0.074 |  |
| nfat_tfpathway | 0.297 | 0.628 | 0.539 | 0.713 | 0.569 | 0.123 | 0.074 |  |
| cmyb_pathway | 0.443 | 0.684 | 0.770 | 1.000 | 0.379 | 0.768 | 0.074 |  |
| p73pathway | 0.443 | 0.610 | 0.608 | 0.749 | 0.509 | 0.323 | 0.074 |  |
| plk3_pathway | 0.087 | 0.132 | 0.069 | 0.119 | 0.112 | 0.127 | 0.085 |  |
| il12_2pathway | 0.467 | 0.718 | 0.770 | 0.867 | 0.540 | 0.347 | 0.085 |  |
| s1p_s1p1_pathway | 0.324 | 0.496 | 0.544 | 0.529 | 0.232 | 0.273 | 0.092 |  |
| epha2_fwdpathway | 0.056 | 0.177 | 0.200 | 0.213 | 0.117 | 0.117 | 0.092 |  |
| p53downstreampathway | 0.443 | 0.645 | 0.681 | 0.749 | 0.262 | 0.598 | 0.092 |  |
| toll_endogenous_pathway | 0.274 | 0.328 | 0.069 | 0.232 | 0.232 | 0.130 | 0.099 |  |
| fra_pathway | 0.223 | 0.204 | 0.469 | 0.529 | 0.140 | 0.312 | 0.101 |  |
| rb_1pathway | 0.443 | 0.628 | 0.539 | 0.751 | 0.601 | 0.199 | 0.118 |  |
| syndecan_3_pathway | 0.202 | 0.229 | 0.112 | 0.232 | 0.213 | 0.086 | 0.131 |  |
| epha_fwdpathway | 0.241 | 0.106 | 0.142 | 0.316 | 0.214 | 0.323 | 0.134 |  |
| hdac_classii_pathway | 0.252 | 0.435 | 0.501 | 0.700 | 0.378 | 0.526 | 0.151 |  |
| atm_pathway | 0.431 | 0.570 | 0.617 | 0.864 | 0.112 | 0.768 | 0.151 |  |
| il6_7pathway | 0.252 | 0.318 | 0.441 | 0.522 | 0.213 | 0.294 | 0.151 |  |
| hedgehog_glipathway | 0.598 | 0.737 | 0.501 | 0.787 | 0.816 | 0.237 | 0.169 |  |
| cd8tcrdownstreampathway | 0.191 | 0.530 | 0.426 | 0.441 | 0.446 | 0.312 | 0.185 |  |
| gmcsf_pathway | 0.691 | 0.685 | 0.705 | 0.749 | 0.540 | 0.656 | 0.185 |  |
| angiopoietinreceptor_pathway | 0.146 | 0.343 | 0.112 | 0.316 | 0.213 | 0.130 | 0.185 |  |
| s1p_s1p2_pathway | 0.475 | 0.561 | 0.392 | 0.474 | 0.443 | 0.550 | 0.196 |  |
| erbb4_pathway | 0.182 | 0.284 | 0.167 | 0.282 | 0.213 | 0.178 | 0.218 |  |
| s1p_s1p5_pathway | 0.452 | 0.570 | 0.532 | 0.316 | 0.262 | 0.493 | 0.218 |  |
| anthraxpathway | 0.143 | 0.059 | 0.189 | 0.202 | 0.140 | 0.255 | 0.218 |  |
| tcrcalciumpathway | 0.368 | 0.645 | 0.338 | 0.710 | 0.608 | 0.463 | 0.218 |  |
| ptp1bpathway | 0.598 | 0.628 | 0.615 | 0.867 | 0.608 | 0.634 | 0.225 |  |
| fgf_pathway | 0.274 | 0.420 | 0.501 | 0.906 | 0.540 | 0.306 | 0.228 |  |
| tcrraspathway | 0.661 | 0.604 | 0.615 | 0.634 | 0.539 | 0.600 | 0.228 |  |
| s1p_s1p4_pathway | 0.652 | 0.628 | 0.617 | 0.700 | 0.540 | 0.600 | 0.228 |  |
| wnt_canonical_pathway | 0.634 | 0.706 | 0.630 | 0.749 | 0.633 | 0.550 | 0.228 |  |
| reg_gr_pathway | 0.715 | 0.981 | 0.611 | 0.794 | 0.608 | 0.634 | 0.228 |  |
| tcrjnkpathway | 0.202 | 0.146 | 0.258 | 0.275 | 0.140 | 0.255 | 0.228 |  |
| cxcr4_pathway | 0.117 | 0.082 | 0.112 | 0.277 | 0.132 | 0.130 | 0.228 |  |
| deltanp63pathway | 0.407 | 0.420 | 0.532 | 0.529 | 0.194 | 0.778 | 0.238 |  |
| ephbfwdpathway | 0.549 | 0.570 | 0.591 | 0.631 | 0.315 | 0.549 | 0.238 |  |
| et_egfrpathway | 0.443 | 0.570 | 0.501 | 0.631 | 0.193 | 0.549 | 0.243 |  |
| hes_heypathway | 0.688 | 0.809 | 0.907 | 0.928 | 0.592 | 0.934 | 0.253 |  |
| nephrin_neph1_pathway | 0.694 | 0.706 | 0.681 | 0.851 | 0.554 | 0.628 | 0.269 |  |
| thrombin_par1_pathway | 0.443 | 0.178 | 0.385 | 0.081 | 0.164 | 0.323 | 0.305 |  |
| pi3kplctrkpathway | 0.719 | 0.809 | 0.571 | 0.928 | 0.963 | 0.507 | 0.305 |  |
| pi3kcipathway | 0.598 | 0.699 | 0.713 | 0.881 | 0.539 | 0.550 | 0.322 |  |
| prlsignalingeventspathway | 0.443 | 0.393 | 0.611 | 0.529 | 0.213 | 0.463 | 0.322 |  |
| telomerasepathway | 0.443 | 0.570 | 0.571 | 0.569 | 0.586 | 0.378 | 0.335 |  |
| erbb1_internalization_pathway | 0.598 | 0.602 | 0.608 | 0.631 | 0.554 | 0.695 | 0.340 |  |
| tcptp_pathway | 0.598 | 0.708 | 0.681 | 0.867 | 0.519 | 0.378 | 0.340 |  |
| syndecan_4_pathway | 0.530 | 0.570 | 0.608 | 0.631 | 0.509 | 0.598 | 0.340 |  |
| integrin4_pathway | 0.274 | 0.276 | 0.190 | 1.000 | 0.258 | 0.294 | 0.340 |  |
| erbb1_receptor_proximal_pathway | 0.600 | 0.598 | 0.611 | 0.718 | 0.586 | 0.605 | 0.367 |  |
| ret_pathway | 0.665 | 0.696 | 0.681 | 0.864 | 0.608 | 0.664 | 0.367 |  |
| netrin_pathway | 0.598 | 0.592 | 0.608 | 0.631 | 0.554 | 0.550 | 0.367 |  |
| glypican_3pathway | 0.274 | 0.284 | 0.302 | 0.202 | 0.164 | 0.294 | 0.373 |  |
| pdgf_pathway | 0.407 | 0.272 | 0.372 | 1.000 | 0.261 | 0.378 | 0.373 |  |
| cxcr3pathway | 0.665 | 0.590 | 0.608 | 0.203 | 0.539 | 0.513 | 0.381 |  |
| arf_3pathway | 0.475 | 0.511 | 0.558 | 0.536 | 0.494 | 0.508 | 0.388 |  |
| erbb1_downstream_pathway | 0.504 | 0.529 | 0.694 | 0.631 | 0.213 | 0.507 | 0.388 |  |
| alk1pathway | 0.297 | 0.204 | 0.258 | 0.202 | 0.117 | 0.277 | 0.388 |  |
| era_genomic_pathway | 0.202 | 0.177 | 0.258 | 0.202 | 0.164 | 0.201 | 0.399 |  |
| p38_mkk3_6pathway | 0.599 | 0.424 | 0.611 | 0.529 | 0.509 | 0.583 | 0.426 |  |
| rapid_gr_pathway | 0.449 | 0.415 | 0.501 | 0.474 | 0.394 | 0.463 | 0.444 |  |
| hedgehog_2pathway | 0.530 | 0.529 | 0.608 | 0.631 | 0.509 | 0.634 | 0.473 |  |
| igf1_pathway | 0.368 | 0.321 | 0.571 | 0.631 | 0.262 | 0.297 | 0.473 |  |
| lis1pathway | 0.611 | 0.592 | 0.617 | 0.536 | 0.294 | 0.549 | 0.473 |  |
| thrombin_par4_pathway | 0.449 | 0.204 | 0.398 | 0.631 | 0.245 | 0.549 | 0.473 |  |
| myc_activpathway | 0.665 | 0.699 | 0.686 | 0.867 | 0.608 | 0.783 | 0.473 |  |
| tgfbrpathway | 0.665 | 0.652 | 0.681 | 0.749 | 0.612 | 0.656 | 0.473 |  |
| a4b7_pathway | 0.688 | 0.746 | 0.611 | 0.773 | 0.682 | 0.549 | 0.473 |  |
| cd8tcrpathway | 0.694 | 0.706 | 0.895 | 0.867 | 0.667 | 0.677 | 0.473 |  |
| p38alphabetapathway | 0.715 | 0.906 | 0.777 | 0.928 | 0.667 | 0.715 | 0.473 |  |
| ephrinbrevpathway | 0.715 | 0.715 | 0.630 | 0.710 | 0.682 | 0.549 | 0.473 |  |
| p75ntrpathway | 0.200 | 0.082 | 0.145 | 0.277 | 0.261 | 0.358 | 0.473 |  |
| amb2_neutrophils_pathway | 0.599 | 0.620 | 0.611 | 0.631 | 0.586 | 0.586 | 0.473 |  |
| erbb_network_pathway | 0.297 | 0.391 | 0.312 | 0.529 | 0.367 | 0.255 | 0.473 |  |
| smad2_3nuclearpathway | 0.715 | 0.684 | 0.770 | 0.749 | 0.608 | 0.768 | 0.473 |  |
| il23pathway | 0.827 | 0.910 | 0.919 | 1.000 | 0.883 | 0.776 | 0.473 |  |
| s1p_meta_pathway | 0.598 | 0.570 | 0.501 | 0.631 | 0.551 | 0.463 | 0.473 |  |
| glypican_2pathway | 0.530 | 0.570 | 0.571 | 1.000 | 0.509 | 0.544 | 0.473 |  |
| cdc42_pathway | 0.719 | 0.766 | 0.895 | 0.943 | 0.539 | 0.882 | 0.474 |  |
| rac1_pathway | 0.667 | 0.706 | 0.630 | 0.536 | 0.608 | 0.549 | 0.476 |  |
| betacatenin_nuc_pathway | 0.873 | 0.966 | 0.880 | 0.991 | 0.898 | 0.780 | 0.508 |  |
| bcr_5pathway | 0.913 | 0.906 | 0.919 | 0.991 | 0.708 | 0.928 | 0.520 |  |
| wnt_noncanonical_pathway | 0.407 | 0.188 | 0.501 | 0.316 | 0.287 | 0.598 | 0.533 |  |
| tcr_pathway | 0.449 | 0.511 | 0.539 | 0.541 | 0.539 | 0.387 | 0.540 |  |
| myc_pathway | 0.598 | 0.590 | 0.611 | 0.652 | 0.554 | 0.598 | 0.543 |  |
| ecadherin_stabilization_pathway | 0.443 | 0.297 | 0.654 | 0.202 | 0.212 | 0.378 | 0.543 |  |
| trail_pathway | 0.665 | 0.748 | 0.675 | 0.867 | 0.667 | 0.613 | 0.543 |  |
| lymphangiogenesis_pathway | 0.876 | 0.910 | 0.842 | 0.864 | 0.694 | 0.656 | 0.543 |  |
| s1p_s1p3_pathway | 0.598 | 0.590 | 0.611 | 0.529 | 0.554 | 0.549 | 0.543 |  |
| foxopathway | 0.443 | 0.570 | 0.615 | 0.541 | 0.313 | 0.603 | 0.543 |  |
| aurora_c_pathway | 0.619 | 0.635 | 0.615 | 0.706 | 0.608 | 0.598 | 0.544 |  |
| vegf_vegfr_pathway | 0.475 | 0.359 | 0.352 | 1.000 | 0.307 | 0.598 | 0.544 |  |
| nfat_3pathway | 0.787 | 0.810 | 0.785 | 0.920 | 0.682 | 0.641 | 0.544 |  |
| ephrinarevpathway | 0.606 | 0.628 | 0.617 | 0.691 | 0.443 | 0.600 | 0.544 |  |
| p38gammadeltapathway | 0.665 | 0.630 | 0.671 | 0.822 | 0.601 | 0.567 | 0.544 |  |
| il27pathway | 0.665 | 0.684 | 0.615 | 0.732 | 0.608 | 0.508 | 0.549 |  |
| glypican_1pathway | 0.715 | 0.706 | 0.730 | 0.892 | 0.633 | 0.789 | 0.549 |  |
| hif2pathway | 0.787 | 0.901 | 0.686 | 0.881 | 0.841 | 0.598 | 0.549 |  |
| p53regulationpathway | 0.734 | 0.909 | 0.815 | 1.000 | 0.876 | 0.788 | 0.549 |  |
| ps1pathway | 0.908 | 0.858 | 0.762 | 0.906 | 0.764 | 0.819 | 0.564 |  |
| p38alphabetadownstreampathway | 0.719 | 0.706 | 0.782 | 0.864 | 0.509 | 0.716 | 0.573 |  |
| alk2pathway | 0.475 | 0.530 | 0.571 | 0.529 | 0.449 | 0.518 | 0.587 |  |
| ilk_pathway | 0.813 | 0.861 | 0.864 | 0.904 | 0.682 | 0.656 | 0.606 |  |
| il8cxcr1_pathway | 0.504 | 0.570 | 0.571 | 0.684 | 0.551 | 0.600 | 0.606 |  |
| a6b1_a6b4_integrin_pathway | 0.241 | 0.146 | 0.316 | 0.316 | 0.185 | 0.359 | 0.606 |  |
| rhoa_pathway | 0.694 | 0.706 | 0.615 | 0.895 | 0.551 | 0.561 | 0.606 |  |
| tap63pathway | 0.504 | 0.570 | 0.514 | 0.529 | 0.514 | 0.508 | 0.611 |  |
| syndecan_pathway | 0.665 | 0.665 | 0.681 | 1.000 | 0.622 | 0.632 | 0.611 |  |
| myc_represspathway | 0.813 | 0.823 | 0.815 | 0.928 | 0.682 | 0.747 | 0.613 |  |
| pdgfrbpathway | 0.812 | 0.769 | 0.708 | 0.580 | 0.688 | 0.643 | 0.614 |  |
| reelinpathway | 0.665 | 0.647 | 0.687 | 0.822 | 0.608 | 0.650 | 0.626 |  |
| il4_2pathway | 0.812 | 0.904 | 0.895 | 0.945 | 0.816 | 0.789 | 0.627 |  |
| ecadherin_keratinocyte_pathway | 0.745 | 0.758 | 0.730 | 0.851 | 0.633 | 0.539 | 0.642 |  |
| il2_stat5pathway | 0.957 | 0.915 | 0.919 | 1.000 | 0.898 | 0.907 | 0.653 |  |
| ajdiss_2pathway | 0.891 | 0.882 | 0.943 | 0.983 | 0.776 | 0.768 | 0.658 |  |
| dnapk_pathway | 0.665 | 0.684 | 0.681 | 0.751 | 0.608 | 0.656 | 0.658 |  |
| hif1_tfpathway | 0.951 | 0.966 | 0.940 | 0.985 | 0.841 | 0.946 | 0.658 |  |
| pi3kcibpathway | 0.699 | 0.706 | 0.667 | 0.749 | 0.682 | 0.641 | 0.673 |  |
| ifngpathway | 0.957 | 0.975 | 0.962 | 0.953 | 0.876 | 0.824 | 0.673 |  |
| erb_genomic_pathway | 0.954 | 0.810 | 0.940 | 0.991 | 0.937 | 0.936 | 0.697 |  |
| alphasynuclein_pathway | 0.297 | 0.205 | 0.426 | 0.119 | 0.164 | 0.315 | 0.698 |  |
| lpa4_pathway | 0.957 | 0.975 | 0.940 | 0.953 | 0.932 | 0.899 | 0.718 |  |
| hdac_classi_pathway | 0.694 | 0.628 | 0.713 | 0.749 | 0.581 | 0.778 | 0.718 |  |
| cd40_pathway | 0.619 | 0.570 | 0.611 | 0.718 | 0.509 | 0.592 | 0.718 |  |
| ncadherinpathway | 0.876 | 0.862 | 0.857 | 0.890 | 0.608 | 0.634 | 0.718 |  |
| ar_pathway | 0.735 | 0.706 | 0.777 | 0.949 | 0.816 | 0.778 | 0.718 |  |
| integrin_a4b1_pathway | 0.827 | 0.862 | 0.854 | 0.906 | 0.816 | 0.868 | 0.718 |  |
| kitpathway | 0.665 | 0.393 | 0.611 | 0.713 | 0.608 | 0.417 | 0.718 |  |
| arf6downstreampathway | 0.665 | 0.609 | 0.611 | 0.599 | 0.527 | 0.561 | 0.718 |  |
| hnf3apathway | 0.913 | 1.000 | 0.532 | 0.971 | 1.000 | 0.507 | 0.718 |  |
| il8cxcr2_pathway | 0.606 | 0.603 | 0.608 | 0.714 | 0.626 | 0.550 | 0.718 |  |
| lkb1_pathway | 0.830 | 0.706 | 0.880 | 0.864 | 0.621 | 0.866 | 0.718 |  |
| circadianpathway | 0.715 | 0.719 | 0.708 | 0.867 | 0.813 | 0.656 | 0.728 |  |
| insulin_pathway | 0.665 | 0.628 | 0.611 | 0.736 | 0.626 | 0.561 | 0.728 |  |
| ceramide_pathway | 0.916 | 0.747 | 0.724 | 0.631 | 0.819 | 0.691 | 0.728 |  |
| arf6_pathway | 0.957 | 0.981 | 0.901 | 1.000 | 0.987 | 0.902 | 0.728 |  |
| il2_pi3kpathway | 0.962 | 0.981 | 0.842 | 0.864 | 0.876 | 0.809 | 0.728 |  |
| vegfr1_pathway | 0.665 | 0.628 | 0.608 | 0.316 | 0.608 | 0.590 | 0.731 |  |
| hdac_classiii_pathway | 0.715 | 0.684 | 0.733 | 0.898 | 0.776 | 0.776 | 0.731 |  |
| rxr_vdr_pathway | 0.715 | 0.706 | 0.708 | 0.822 | 0.667 | 0.656 | 0.731 |  |
| ecadherin_nascentaj_pathway | 0.944 | 0.974 | 0.907 | 1.000 | 0.928 | 0.904 | 0.742 |  |
| smad2_3pathway | 0.876 | 0.897 | 0.644 | 0.906 | 0.835 | 0.776 | 0.746 |  |
| integrin1_pathway | 0.580 | 0.590 | 0.558 | 0.864 | 0.527 | 0.598 | 0.765 |  |
| betacatenin_deg_pathway | 0.954 | 0.962 | 0.940 | 0.985 | 0.928 | 0.897 | 0.780 |  |
| rac1_reg_pathway | 0.891 | 0.897 | 0.842 | 0.928 | 0.876 | 0.788 | 0.782 |  |
| notch_pathway | 0.688 | 0.604 | 0.630 | 0.631 | 0.562 | 0.598 | 0.788 |  |
| integrin2_pathway | 0.952 | 0.935 | 0.919 | 0.982 | 0.932 | 0.866 | 0.800 |  |
| hif1apathway | 0.865 | 0.918 | 0.864 | 0.928 | 0.835 | 0.743 | 0.800 |  |
| retinoic_acid_pathway | 0.603 | 0.613 | 0.646 | 0.822 | 0.586 | 0.592 | 0.820 |  |
| avb3_opn_pathway | 0.952 | 0.810 | 0.842 | 0.713 | 0.876 | 0.778 | 0.844 |  |
| p38_mk2pathway | 0.952 | 0.792 | 0.919 | 0.684 | 0.858 | 0.904 | 0.851 |  |
| bmppathway | 0.787 | 0.925 | 0.901 | 0.928 | 0.682 | 0.789 | 0.851 |  |
| integrin3_pathway | 0.952 | 0.918 | 0.919 | 0.982 | 0.928 | 0.904 | 0.869 |  |
| ranbp2pathway | 0.715 | 0.628 | 0.713 | 0.710 | 0.626 | 0.656 | 0.882 |  |
| hnf3bpathway | 0.952 | 0.901 | 0.940 | 0.982 | 0.841 | 0.876 | 0.882 |  |
| cone_pathway | 0.965 | 0.981 | 0.940 | 1.000 | 0.972 | 0.768 | 0.886 |  |
| rhodopsin_pathway | 0.981 | 0.981 | 0.962 | 1.000 | 0.972 | 0.809 | 0.886 |  |
| rhoa_reg_pathway | 0.511 | 0.369 | 0.446 | 0.441 | 0.355 | 0.737 | 0.886 |  |
| hivnefpathway | 0.913 | 0.862 | 0.770 | 0.928 | 1.000 | 0.737 | 0.891 |  |
| tnfpathway | 0.665 | 0.602 | 0.615 | 0.541 | 0.587 | 0.507 | 0.891 |  |
| arf6_traffickingpathway | 0.993 | 0.981 | 1.000 | 1.000 | 0.985 | 1.000 | 0.891 |  |
| syndecan_2_pathway | 0.968 | 0.981 | 0.681 | 1.000 | 1.000 | 0.648 | 0.904 |  |
| mtor_4pathway | 0.957 | 0.935 | 0.854 | 0.881 | 0.963 | 0.656 | 0.909 |  |
| il3_pathway | 0.811 | 0.712 | 0.815 | 0.906 | 0.701 | 0.876 | 0.925 |  |
| syndecan_1_pathway | 0.812 | 0.652 | 0.718 | 0.582 | 0.701 | 0.656 | 0.927 |  |
| avb3_integrin_pathway | 0.665 | 0.590 | 0.730 | 0.631 | 0.592 | 0.656 | 0.943 |  |
| caspase_pathway | 0.801 | 0.719 | 0.762 | 0.906 | 0.701 | 0.776 | 0.943 |  |
| cdc42_reg_pathway | 0.985 | 0.981 | 0.962 | 1.000 | 0.977 | 0.955 | 0.967 |  |
| nectin_pathway | 0.993 | 0.981 | 0.962 | 0.864 | 0.963 | 0.928 | 0.967 |  |
| upa_upar_pathway | 0.993 | 0.981 | 1.000 | 0.982 | 0.963 | 1.000 | 0.967 |  |
| integrin5_pathway | 0.972 | 0.981 | 0.962 | 1.000 | 0.985 | 0.952 | 0.970 |  |
| wnt_signaling_pathway | 0.993 | 0.981 | 1.000 | 1.000 | 0.937 | 1.000 | 1.000 |  |
| ephrina_ephapathway | 1.000 | 1.000 | 1.000 | 1.000 | 1.000 | 1.000 | 1.000 |  |
| nfkappabcanonicalpathway | 1.000 | 1.000 | 1.000 | 1.000 | 1.000 | 1.000 | 1.000 |  |
| glypicanpathway | 1.000 | 1.000 | 1.000 | 1.000 | 1.000 | 1.000 | 1.000 |  |
| hnf3pathway | 1.000 | 1.000 | 1.000 | 1.000 | 1.000 | 1.000 | 1.000 |  |
| ephrinb_ephbpathway | 1.000 | 1.000 | 1.000 | 1.000 | 1.000 | 1.000 | 1.000 |  |
| integrin_cs_pathway | 1.000 | 1.000 | 1.000 | 1.000 | 1.000 | 1.000 | 1.000 |  |
| aurora_kinase_pathway | 1.000 | 1.000 | 1.000 | 1.000 | 1.000 | 1.000 | 1.000 |  |
| plk_pathway | 1.000 | 1.000 | 1.000 | 1.000 | 1.000 | 1.000 | 1.000 |  |
| p63pathway | 1.000 | 1.000 | 1.000 | 1.000 | 1.000 | 1.000 | 1.000 |  |
| thrombin_pathway | 1.000 | 1.000 | 1.000 | 1.000 | 1.000 | 1.000 | 1.000 |  |
| faspathway | 0.957 | 0.975 | 0.962 | 0.991 | 0.816 | 1.000 | 1.000 |  |
| gr_signal_pathway | 1.000 | 1.000 | 1.000 | 1.000 | 1.000 | 1.000 | 1.000 |  |
| insulin_glucose_pathway | 1.000 | 1.000 | 1.000 | 1.000 | 1.000 | 1.000 | 1.000 |  |
| integrin_a9b1_pathway | 0.532 | 0.570 | 0.586 | 0.631 | 0.539 | 0.590 | 1.000 |  |
| alk1_2pathway | 1.000 | 1.000 | 1.000 | 1.000 | 1.000 | 1.000 | 1.000 |  |
| il8_pathway | 1.000 | 1.000 | 1.000 | 1.000 | 1.000 | 1.000 | 1.000 |  |
| nfkappabalternativepathway | 1.000 | 1.000 | 1.000 | 1.000 | 1.000 | 1.000 | 1.000 |  |
| ecadherin_1_pathway | 1.000 | 1.000 | 1.000 | 1.000 | 1.000 | 1.000 | 1.000 |  |
| androgen_pathway | 1.000 | 1.000 | 1.000 | 1.000 | 1.000 | 1.000 | 1.000 |  |
| il5_pathway | 1.000 | 1.000 | 1.000 | 1.000 | 1.000 | 1.000 | 1.000 |  |
| integrin7_pathway | 1.000 | 1.000 | 1.000 | 1.000 | 1.000 | 1.000 | 1.000 |  |
| nfkappabatypicalpathway | 1.000 | 1.000 | 1.000 | 1.000 | 1.000 | 1.000 | 1.000 |  |
| botulinumtoxinpathway | 1.000 | 1.000 | 1.000 | 1.000 | 1.000 | 1.000 | 1.000 |  |
| pi3kciaktpathway | 0.965 | 0.981 | 0.940 | 0.904 | 0.922 | 0.772 | 1.000 |  |
| plk2_4pathway | 1.000 | 1.000 | 1.000 | 1.000 | 1.000 | 1.000 | 1.000 |  |

**Reference:**

1. Pellegrino R, Calvisi DF, Ladu S, et al.: **Oncogenic and tumor suppressive roles of polo-like kinases in human hepatocellular carcinoma.** *Hepatology (Baltimore, Md.)* 2010, **51**:857-68.

2. Whittaker S, Marais R, Zhu AX: **The role of signaling pathways in the development and treatment of hepatocellular carcinoma.** *Oncogene* 2010, **29**:4989-5005.

3. Itoh S: **Role of Expression of Focal Adhesion Kinase in Progression of Hepatocellular Carcinoma**. *Clinical Cancer Research* 2004, **10**:2812-2817.

4. Liu P: **Activation of NF-kappaB, AP-1 and STAT transcription factors is a frequent and early event in human hepatocellular carcinomas**. *Journal of Hepatology* 2002, **37**:63-71.

5. Ribatti D, Marzullo A, Gentile A, et al.: **Erythropoietin/erythropoietin-receptor system is involved in angiogenesis in human hepatocellular carcinoma.** *Histopathology* 2007, **50**:591-6.
